# Conditional generation of real antigen-specific T cell receptor sequences

**DOI:** 10.1038/s42256-025-01096-6

**Published:** 2025-09-08

**Authors:** Dhuvarakesh Karthikeyan, Sarah N. Bennett, Amy G. Reynolds, Benjamin G. Vincent, Alex Rubinsteyn

**Affiliations:** 1 Personalized Immunotherapy Research Lab, University of North Carolina at Chapel Hill, Chapel Hill, NC USA; 2 Curriculum in Bioinformatics and Computational Biology, University of North Carolina at Chapel Hill, Chapel Hill, NC USA; 3 Computational Medicine Program, UNC School of Medicine, University of North Carolina at Chapel Hill, Chapel Hill, NC USA; 4 Department of Biochemistry and Biophysics, UNC School of Medicine, University of North Carolina at Chapel Hill, Chapel Hill, NC USA; 5 Department of Microbiology and Immunology, University of North Carolina, Chapel Hill, Chapel Hill, NC USA; 6 Lineberger Comprehensive Cancer Center, UNC School of Medicine, University of North Carolina at Chapel Hill, Chapel Hill, NC USA; 7 Division of Hematology, Department of Medicine, UNC School of Medicine, University of North Carolina at Chapel Hill, Chapel Hill, NC USA

**Keywords:** Machine learning, Adaptive immunity

## Abstract

Despite recent advances in T cell receptor (TCR) engineering, designing functional TCRs against arbitrary targets remains challenging due to complex rules governing cross-reactivity and limited paired data. Here we present TCR-TRANSLATE, a sequence-to-sequence framework that adapts low-resource machine translation techniques to generate antigen-specific TCR sequences against unseen epitopes. By evaluating 12 model variants of the BART and T5 model architectures, we identified key factors affecting performance and utility, revealing discordances between these objectives. Our flagship model, TCRT5, outperforms existing approaches on computational benchmarks, prioritizing functionally relevant sequences at higher ranks. Most significantly, we experimentally validated a computationally designed TCR against Wilms’ tumour antigen, a therapeutically relevant target in leukaemia, excluded from our training and validation sets. Although the identified TCR shows cross-reactivity with pathogen-derived peptides, highlighting limitations in specificity, our work represents the successful computational design of a functional TCR construct against a non-viral epitope from the target sequence alone. Our findings establish a foundation for computational TCR design and reveal current limitations in data availability and methodology, providing a framework for accelerating personalized immunotherapy by reducing the search space for novel targets.

## Abstract (web summary)

TCR-TRANSLATE, a deep learning framework adapting machine translation to immune design, demonstrates the successful generation of a functional T cell receptor sequence for a cancer epitope from the target sequence alone.

## Main

T cells are a subset of immune cells that use stochastically generated, highly specific pattern recognition receptors called T cell receptors (TCRs) to identify cells presenting ‘non-self’ peptides at the cell surface. This immune surveillance relies on a diverse repertoire of TCRs recognizing cognate peptides presented on major histocompatibility complexes (MHCs), creating a network of TCR:peptide-MHC (pMHC) specificities that collectively mediate self–non-self discrimination with single amino acid precision^1^. T cell-based therapies including CAR-T, engineered TCRs and TCR bispecifics have shown durable treatment of chronic infections^2^, autoimmune diseases^3^ and even solid tumours^4^. A critical bottleneck in their development is the identification of specific and self-tolerant TCRs, which rely on laborious and low-yield in vitro TCR discovery platforms^5^. In silico methods to decipher the mapping between TCRs and pMHCs stand to transform precision immunotherapies by operationalizing a potent mechanism of functionally deleting cells at the subprotein resolution.

Experimentally, some individual TCRs have been shown to recognize up to one million unique peptides^6^, and vice versa^7,8^. However, learning this many-to-many mapping is severely confounded by sparse and biased paired data, with most antigen-specific TCRs being identified in the context of only a few, well-studied diseases^9^. Current approaches in modelling antigen specificity frame the problem as a binary classification task^10^ with limited utility in TCR design^11^. Earlier generative models focused on TCR redesign given known antigen-specific repertoires^12^ or unconditional TCR generation that statistically approximates natural repertoires^13,14^ in the aggregate. Recently, conditional generation methods have shown promise, using convolutional neural networks–long short-term memory network architectures to generate TCRs for known antigens^15^. Although we introduced an autoregressive transformer architecture for this problem^16^ alongside others^17^ and related research has since emerged^18^, a deep understanding of the real-world utility of TCR generation remains elusive.

**Fig. 1:**
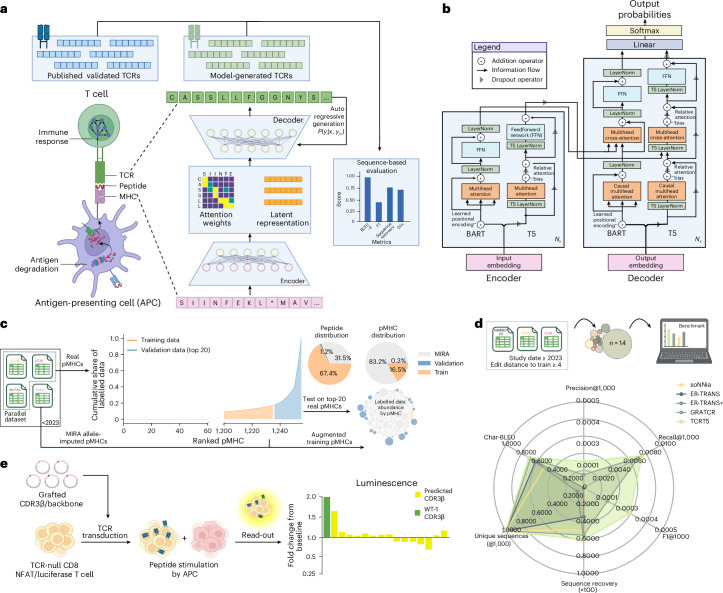
Overview of TCR-TRANSLATE. **a**, Casting antigen-specific TCR design as a seq2seq task. We make use of an encoder–decoder abstraction to process pMHC sequence information and autoregressively sample target-conditioned CDR3β sequences. **b**, Specific architecture of TCRBART and TCRT5. Transformer architecture juxtaposing BART and T5 encoder and decoder layers, highlighting key operations to the residual stream, inspired by Vaswani et al. (2017)^60^. **c**, Dataset creation. Given the severe data sparsity, the top-20 pMHCs from IEDB, VDJdb and McPAS (by known TCRs) were withheld as validation, whereas the remainder were used for training with allele-imputed pMHCs from MIRA. **d**, In silico benchmark performance of TCRT5 and publicly available methods. Overview of benchmark dataset creation (*n* = 14) and performance radar plot are shown with the averaged metrics across pMHCs. soNNia model’s unconditional metrics are averaged over 1,000 simulation runs. **e**, Illustrative diagram of the in vitro validation pipeline of the generated CDR3β sequences using NFAT-associated luciferase expression for T cell activation-induced luminescence. Panels **a**, **c**, **d** and **e** created with BioRender.com.

Following our initial proof of concept^16^, we adopted the formulation of the TCR reactivity problem as a sparse sequence-to-sequence (seq2seq) task (Fig. 1a), introducing TCRBART and TCRT5, two encoder–decoder transformer models based on the BART^19^ and T5 (ref. ^20^) architectures (Fig. 1b and Supplementary Note A.1). Here, to directly address the issues of sparse parallel data comprising source–target sequence pairs, we investigate a handful of techniques from low-resourced machine translation^21^ for this task. One particularly effective approach leverages the reflexive nature of sequence co-dependencies between source–target pairs by jointly learning a bidirectional mapping^22,23^, sharing representations and aligning latent spaces across both sequences^24^. However, to the best of our knowledge, these approaches have not been applied to the functional protein design space.

In this work, we systematically trained 12 TCRBART and TCRT5 variants using a handful of these low-resource techniques and assessed the fidelity of their generations. We constructed a validation dataset comprising the top-20 pMHCs with the most known cognate TCRs (Fig. 1c), forfeiting their inclusion in the training data to maximize exact sequence matches during validation. Finally, we benchmark our flagship TCRT5 model on a robust test set against existing methods (Fig. 1d) and validate our model in vitro, sampling a complementarity determining region (CDR3β) sequence not seen during training that shows functional activity against a challenging target (Fig. 1e). Our results demonstrate the potential of seq2seq modelling for generating antigen-specific TCRs and the current limitations imposed by severely constrained data (Supplementary Note A.2).

## Results

**Fig. 2:**
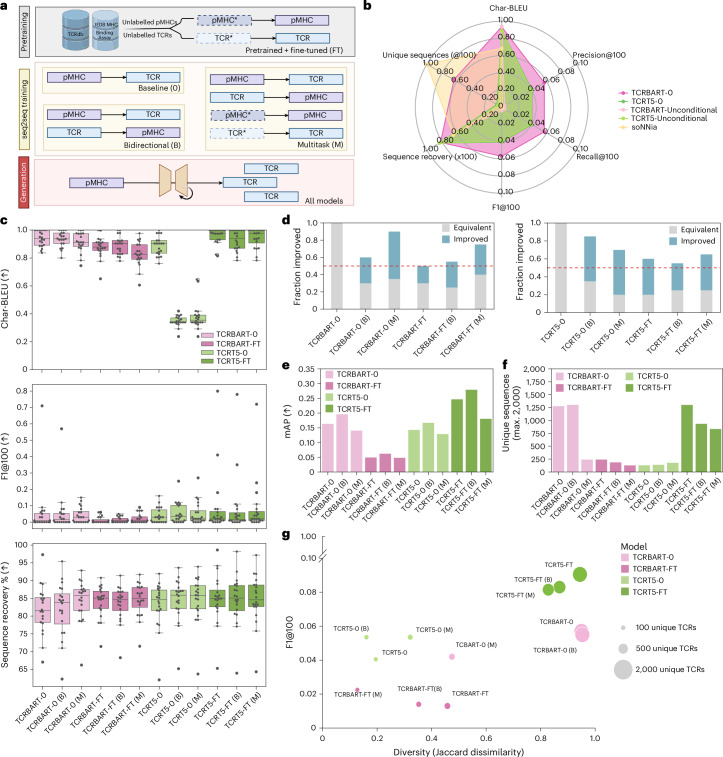
Multitask training increases accuracy and decreases diversity metrics. **a**, Diagram outlining pretraining, seq2seq training/fine-tuning and their common generation scheme (inference). **b**, Radar plot showing the performance of TCRBART-0 and TCRT5-0 and their unconditional variants TCRBART-Unconditional/TCRT5-Unconditional against the averaged metrics over 1,000 simulations of the statistical soNNia generative model^14^. **c**, TCR-TRANSLATE accuracy metrics. Box and whisker plots show the median, quartile and individual contributions of each of the validation pMHCs (*n* = 20) for Char-BLEU, F1@100 and native sequence recovery. Whiskers extend to 1.5× the interquartile range. **d**, Fraction of pMHC F1@100 scores that remain equivalent to or greater than the baseline models (TCRBART-0 and TCRT5-0). Red line marks the 50% point, indicating no gain in performance compared with the baseline. **e**, Model calibration as measured by mAP across pMHCs calculated using sequence-likelihood-based rank per model. **f**, Bar plot of global diversity calculated as the total number of unique sequences across pMHCs (20 × 100 = 2,000 (maximum)). **g**, Scatter plot summarizing the model performance on accuracy and diversity metrics. Accuracy is taken as the mean F1@100 score and diversity is shown in terms of the total number of unique sequences generated (size of each data point) as well as the mean pairwise Jaccard dissimilarity scores across pMHCs (*x* axis). Panel **a** created with BioRender.com.

For our experiments, we considered three different training schemes for TCRBART and TCRT5 each, stratified by pretraining status for a total of 12 model variants. All models were evaluated on CDR3β sequence generation (Fig. 2a). The baseline models (TCRBART-0 and TCRT5-0) were trained on pMHC→TCR generation without pretraining. Bidirectional models (TCRBART-0 (B) and TCRT5-0 (B)) were trained on both directions (pMHC↔TCR), and multitask models additionally included a masked language modelling term for both TCR and pMHC reconstructions (TCRBART-0 (M) and TCRT5-0 (M)). Similarly, six models were pretrained on reconstruction and then fine-tuned using the same learning tasks to add TCRBART-FT, TCRBART-FT (B), TCRBART-FT (M), TCRT5-FT, TCRT5-FT (B) and TCRT5-FT (M). All sequence sampling was done using the beam search decoding algorithm (Supplementary Note A.3), a heuristic algorithm that generates sequences of high probability found to be effective for this task^16–18^.

### Conditional generation outperforms unconditional generation

Before comparing our various training techniques, we first sought to run hyperparameter optimization (Supplementary Note A.4) and then calibrate our metrics by benchmarking conditional models *P*(TCR∣pMHC) against unconditional generation *P*(TCR). To determine the advantage of input conditioning, we evaluated our baseline conditional models TCRBART-0 and TCRT5-0 on a subset of our metrics (Supplementary Note A.5). As our unconditional baseline, we used soNNia’s ‘Ppost’^14^, a generative model that extends the immune-specific process of generating T and B cell receptors known as V(D)J recombination to include thymic selection, sampling a TCR distribution closer to what is observed in the periphery, independent of the target antigen. Additionally, to investigate our training set composition’s impact on the validation performance, we evaluated sequences from TCRBART-0 and TCRT5-0 derived in an input-free manner (TCRBART-Unconditional and TCRT5-Unconditional). As expected, we found that conditioning on the epitope yielded gains on all metrics, except for global diversity (Fig. 2b). Surprisingly, TCRT5-Unconditional achieved non-zero F1 scores, revealing high-likelihood training CDR3β sequences in the validation set.

### Multitask training increases accuracy metrics and decreases diversity metrics of generated sequences

**Extended Data Fig. 1:**
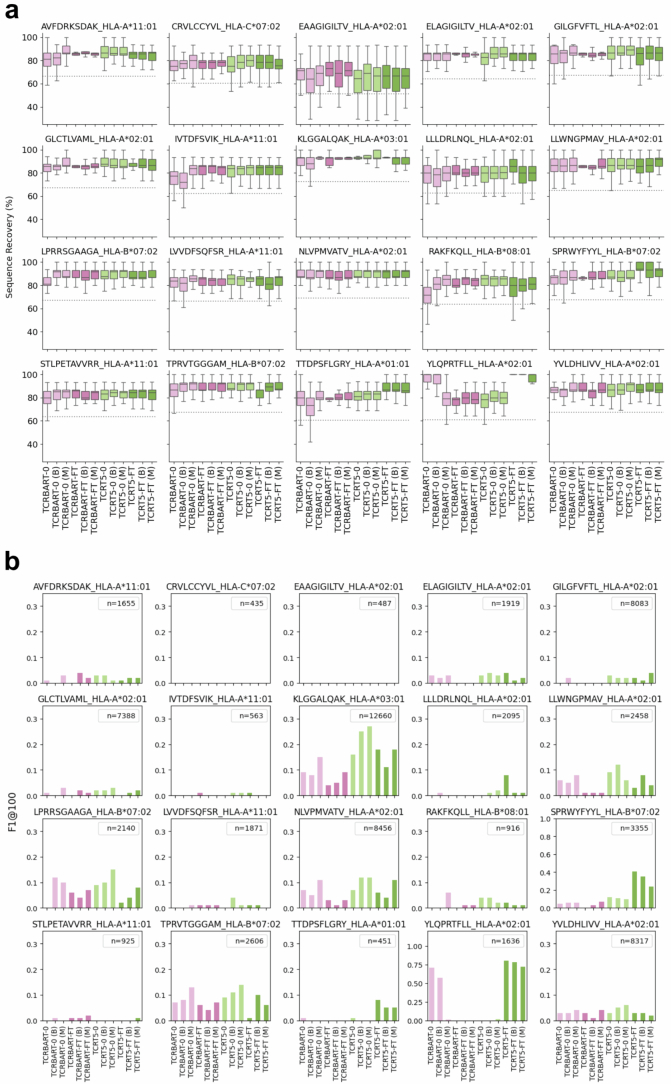
Atomic metrics. (**a**) Box and whisker plot showing median and quartile ranges for individual translation sequence recoveries (*n* = 100) per model per pMHC. Whiskers extend to 1.5× IQR. Dotted lines show the mean sequence recovery of 10k soNNia unconditional sequences computed on the true cognate paratope sequences. (**b**) Bar plot showing F1@100 score per model and pMHC. Each subplot is demarcated with the number of reference CDR3*β*s in the top right corner.

After confirming the utility of target conditioning, we trained the additional TCRBART and TCRT5 variants and found that no single model outperformed the others on all metrics across all pMHCs (Extended Data Fig. 1a,b). For example, although sequence recoveries increased for bidirectional and multitask variants, their Char-BLEU scores decreased (Fig. 2c). For the F1 score, some models excelled on a small subset of examples and others showed marginal improvements across a broader set, reflected in the divergent mean and median scores. Reassuringly, all the training procedures maintained or improved the F1 performance for over 50% of validation pMHCs over the baseline (Fig. 2d). Using the mean average precision (mAP) to assess calibration (ranking of observed binders), we found that the bidirectional models outperformed the baseline and both outperformed the multitask variants (Fig. 2e). Diversity metrics, however, revealed a decline in unique sequences generated across pMHCs, going from the baseline models to the bidirectional and multitask ones. This was most evident for TCRBART-0 (M), which retained strong performance despite a drop of over 80% in unique generations (Fig. 2f), highlighting the importance of using both metrics to represent performance.

**Extended Data Fig. 2:**
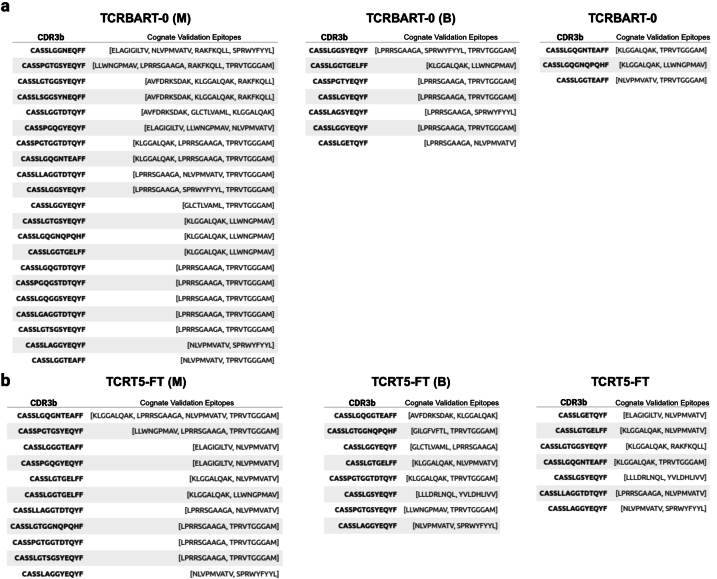
Multitask models sample more known validated polyspecific TCR sequences. (**a**) Subset of TCRBART-0 generations across model variants that are known binders to more than one validation pMHC (may be from the same disease context). (**b**) Subset of TCRT5-FT generations across model variants that are known binders to more than one validation pMHC (may be from the same disease context). Each row is an individual CDR3*β* sequence that was generated for and found in the experimentally validated set of reference TCRs for the listed validation pMHCs.

To holistically characterize the models, we visualized performance on a diversity–accuracy biaxial plot. Although pretraining and fine-tuning pushed the pareto front for the TCRT5-FT variants, we observed the opposite effect in the TCRBART-FT models (Fig. 2g and Supplementary Note A.6). Since both TCRBART-FT and TCRT5-0 generated less than 10% of the maximum number of unique sequences, with average Jaccard dissimilarities of less than 0.5, we selected the TCRBART-0 and TCRT5-FT variants as the best BART and T5 models, respectively, and restrict further analyses to these models. Interestingly, the differences between the baseline, bidirectional and multitask models of TCRBART-0 and TCRT5-FT were less obvious. Crucially, the fact remained that the bidirectional and multitask model variants generated fewer unique sequences and still improved performance. When we examined the generated sequences, we saw that many CDR3β sequences repeatedly sampled across pMHCs were known binders to multiple validation examples (Extended Data Fig. 2).

### Multitask models preferentially sample polyspecific CDR3β sequences

**Fig. 3:**
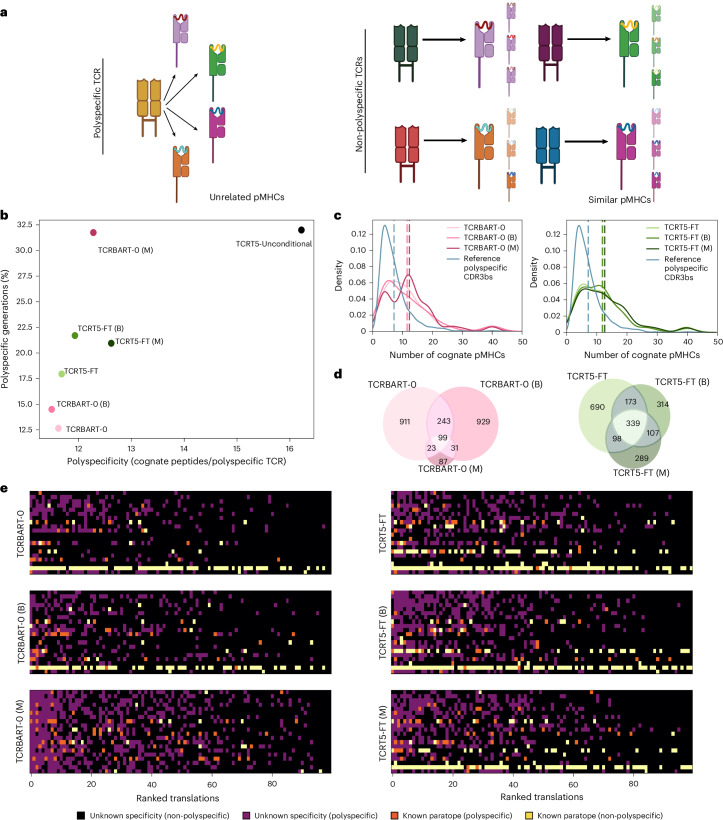
Multitask training promotes degenerate sampling of polyspecific TCRs. **a**, Diagram showing polyspecific TCRs binding different, unrelated pMHCs juxtaposed against regular TCRs sharing a more conserved cross-reactivity profile. **b**, Scatter plot of the number of polyspecific generations as a percentage and mean polyspecificity (number of distinct peptides) of the polyspecific TCRs per model is shown. **c**, Distribution of TCR polyspecificity across the parallel data and model generations. Density plot of cognate peptide counts for polyspecific TCRs aggregated from the combined training and validation set (reference CDR3β sequences) and the model variants per class. **d**, Venn diagrams of translation overlaps for TCRBART-0 and TCRT5-FT model variants. **e**, TCRBART-0 and TCRT5-FT sample polyspecific and known binders with higher sequence likelihoods than those of unknown specificities. Discrete heat maps in which rows indicate individual validation pMHCs, columns indicate the translation rank and colour indicates known binding and polyspecificity status shown for TCRBART-0 and TCRT5-FT variants. Colour/intensity reflects the increasing functional utility of a particular translation as the product of its specificity and known binding status. Panel **a** created with BioRender.com.

Although cross-reactivity is an essential component of the TCR repertoire, a recent study defines distinctly ‘polyspecific’ TCRs (Fig. 3a) as those with higher generation probabilities, specific V/J gene preferences, shared CDR3s across individuals and reactivity to multiple unrelated peptides^25^. Given the improbable nature in which the multitask models maintained competitive performance with a fraction of the diversity, we ventured to qualify the translations’ polyspecificity status. Since our models lack V/J and individual-level context, we employ an ‘ML-centric’ definition to identify CDR3β sequences from our training set appearing in multiple disease contexts and binding more than two epitopes (*n* = 915). We found that not only did the multitask models generate more polyspecific CDR3β sequences (*P* < 0.01 for both architectures; Fisher’s exact test), but that their mean polyspecificity, as defined by the number of cognate epitopes, increased too (Fig. 3b,c). In fact, we observed a strong inverse correlation between the number of polyspecific TCRs and the unique sequences generated (Pearson’s *r*: –0.957), with a high level of sequence sharing between models (Fig. 3d).

**Extended Data Fig. 3:**
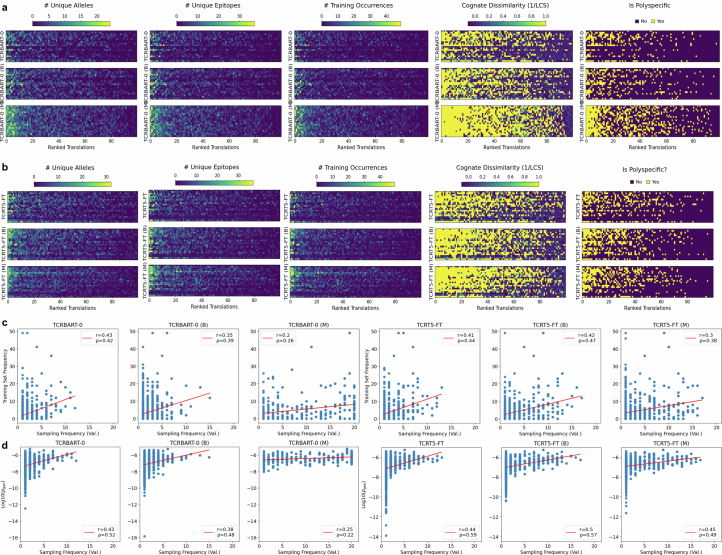
Exploring polyspecificity vs. training set statistics across baseline, bidirectional, and multitask model variants. (**a**) Heat map of ranked TCRBART-0 translations across pMHCs coloured by number of known alleles, known epitopes, training set frequency, epitope dissimilarity (measured as the reciprocal of the longest common substring (LCS)), and membership status in the 915 polyspecific TCRs. (**b**) Analogous heat map as panel ‘a’ but for TCRT5-FT generations. (**c**) Correlation plots for TCRBART-0 and TCRT5-FT model generations and training set occurrence. Line of best fit is shown in red. Pearson’s *r* and Spearman’s ρ are also provided for each model. (**d**) Correlation plots for TCRBART-0 and TCRT5-FT log[*p*_gen_] and model generation frequency. Line of best fit shown in red. Summary statistics are provided as well.

To determine if the models were mimicking CDR3β sequences seen most during training at similar frequencies, we examined the translations’ rank order against potentially explanatory variables such as polyspecificity, number of cognate epitopes/alleles and training set incidence. We found that although the highly ranked sequences were more common in the training set, they also had more dissimilar known cognate epitopes, suggesting robustness in capturing polyspecificity (Extended Data Fig. 3a,b). Multitask models showed weaker correlations between the sampling frequency and both training set occurrence and generation probabilities compared with their baseline and bidirectional counterparts, suggesting that they capture polyspecificity beyond simple memorization (Extended Data Fig. 3c,d).

Since our validation set comprises highly immunogenic viral peptides known to be the targets of polyspecific TCRs^25^, we checked if our F1 performance could be explained solely by polyspecific generations. Although the models sampled polyspecific sequences at higher ranks, we found that baseline models sampled both more non-polyspecific sequences overall and more non-polyspecific true-positive binders (Fig. 3e). Given our desire for a model that generates CDR3β sequences for rare epitopes, we find polyspecific TCR generation a potentially misleading avenue for metric hacking, misrepresenting true usefulness. Thus, although the bidirectional and multitask models show promise in increasing accuracy through self-consistency for the receptor–ligand design problem, we note that their utility may be limited for real-world scenarios. We, therefore, select TCRT5-FT as our flagship model (Supplementary Note A.7) and, henceforth, refer to it simply as TCRT5.

### TCRT5 generates real unseen antigen-specific CDR3β sequences

**Fig. 4:**
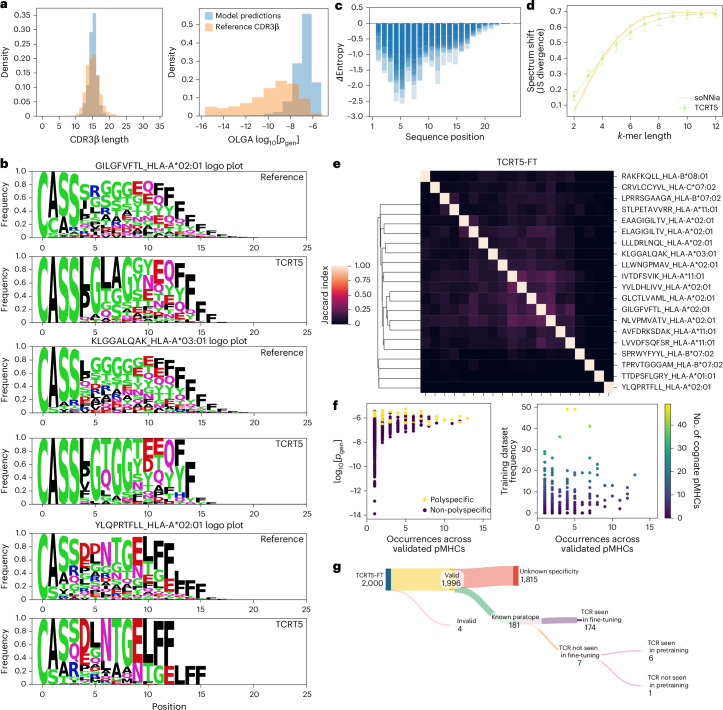
Qualitative assessment of TCRT5. **a**, Repertoire-level features of reference (validation target sequences) and generated CDR3β sequences. TCRT5 captures the tails of the CDR3β length distribution but preferentially samples sequences at the right tail of OLGA generation probabilities. **b**, Sequence logo plots showing the decrease in sequence diversity position across the generated and reference CDR3β sequences for three canonical pMHCs (GILGFVFTL (influenza A), KLGGALQAK (CMV) and YLQPRTFLL (SARS-CoV2)). **c**, Generated sequences experience a decrease in Shannon entropy for nearly all positions compared with the reference sequences across all pMHCs. Bar plots for individual pMHCs are overlaid on one another. **d**, Plot of *k*-mer spectrum shift, showing the JS divergence between the generated and reference sequences. Mean JS divergences for soNNia generations for 100 sequences sampled per pMHC across 100 simulations are shown for reference. Error bars mark the mean and one standard deviation across validation pMHCs (*n* = 20). **e**, Heat map of Jaccard index scores, showing the generated sequence co-occurrence across different pMHC pairs. **f**, TCRT5 repeats sequences across pMHCs in line with biological probabilities and is robust to training set abundance. Scatter plot visualizing the occurrence across pMHCs with OLGA *p*_gen_, polyspecificity and training set frequency. **g**, TCRT5 generates experimentally validated antigen-specific CDR3β sequences unseen during training. Sankey diagram showing the validity (non-zero OLGA *p*_gen_), known antigen specificity status and training set membership of the generated sequences across the validation pMHCs.

**Extended Data Fig. 4:**
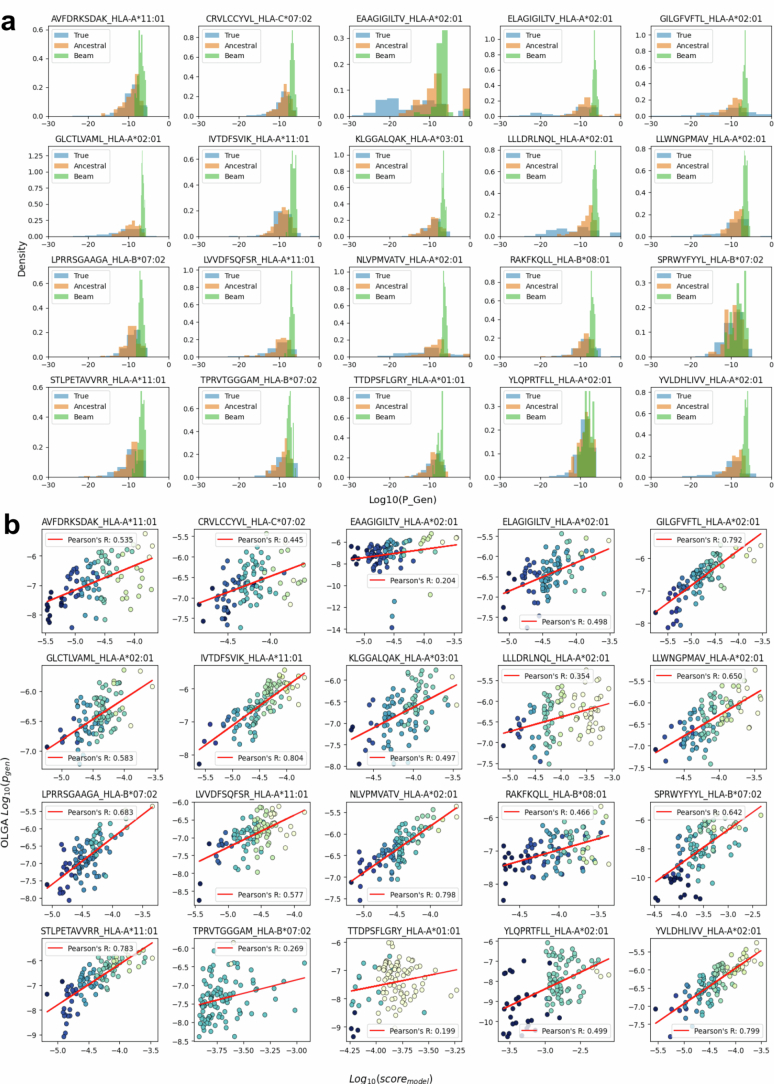
CDR3*β* embeddings highlight reduction in sampled TCR space. PCA dimensionality reduction of embeddings generated by sequence based methods are shown for: (**a**) TCR-BERT (**b**) catELMo^61^ (**c**) TCR2vec^62^. Red points indicate sequences generated by TCRT5, gray corresponds to reference translations, and blue points are soNNia generated sequences. Reference TCRs are downsampled to 200 sequences and 100 background sequences are shown.

**Extended Data Fig. 5:**
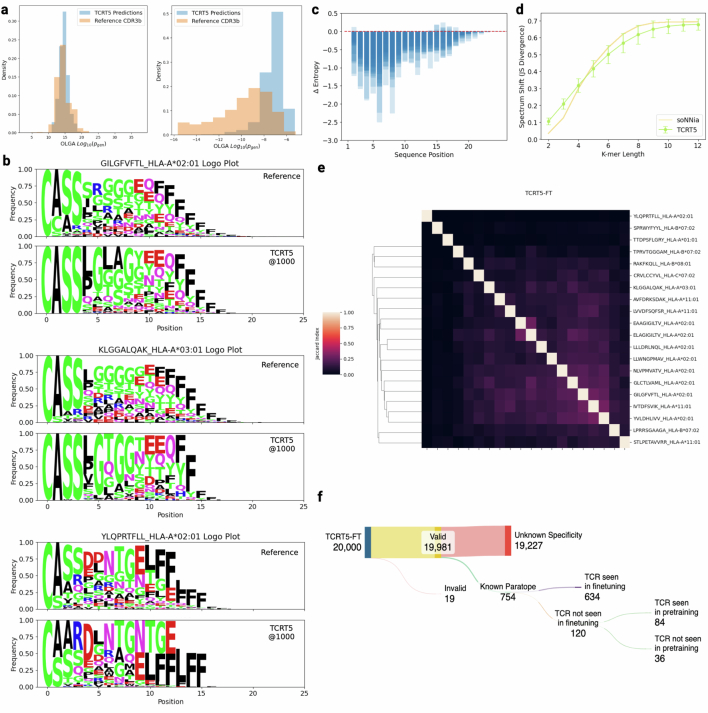
TCRT5 sequence likelihoods. (**a**) Histograms showing the OLGA *p*_gen_ values for the reference CDR3*β*s as well as those generated by beam search and ancestral sampling methods. (**b**) Correlation plots showing the model scores (model sequence likelihoods) against the biophysical OLGA *p*_gen_. Axes are log10 scaled. Red line is the best fit line with associated Pearson’s r.

Having selected TCRT5 for its superior accuracy, diversity and minimal reliance on polyspecific TCRs, we proceeded to understand the model in a more qualitative manner. TCRT5 captures CDR3β lengths with a slight decrease in spread (mean, 14.6; s.d., 1.2) compared with the reference set (mean, 14.5; s.d., 2.0). However, the sampled sequences had a substantially higher generation probability as determined by OLGA^26^, log[*p*_gen_] (mean, –7.04; s.d., 0.85) than the reference (mean, –9.83; s.d., 2.356), indicating that TCRT5 was missing lower-probability sequences (Fig. 4a). This reduction in repertoire diversity was also captured by various sequence embedding models (Extended Data Fig. 4a–c). To determine whether this effect stemmed from our choice of decoding algorithm rather than the model’s weights, we compared the *p*_gen_ values from beam search and ancestral sampling against reference CDR3β sequences and found that beam search shifted the distribution towards higher biological likelihood than ancestral and reference (Extended Data Fig. 5a). Interestingly, we found that these *p*_gen_ values correlated with the model log-likelihood scores (Extended Data Fig. 5b).

Sequence logo plots of the cognate sequences for canonical epitopes GILGFVFTL (influenza A), KLGGALQAK (cytomegalovirus) and YLQPRTFLL (SARS-CoV2) revealed a noticeable decrease in sequence diversity, particularly near the start of the sequence (Fig. 4b). This loss of entropy was quantified using the positional *Δ*entropy value, which showed the greatest loss in entropy around position 5 (Fig. 4c), probably due to the bias of starting sequences with the ‘CASS’ motif. Additionally, we found that soNNia better matched the reference *k*-mer patterns at short lengths, whereas TCRT5 matched better for medium *k*-mer lengths with both converging at the longer *k*-mer lengths (Fig. 4d).

Next, to determine TCRT5’s input specificity, we computed the Jaccard index to assess the overlap between translation sequences across pMHCs (Fig. 4e) and found sequences with high similarity clustered together, such as melanoma antigens EAAGIGILTV and ELAGIGILTV, though more data are required to determine to what extent this generalizes. To check for the correlates of sampling occurrence across validation pMHCs, we compared the generation probabilities, polyspecificity and training set frequency, and found that higher generation probability sequences were more frequently sampled, with no clear correlation between the training frequency and increased sampling (Fig. 4f).

**Extended Data Fig. 6:**
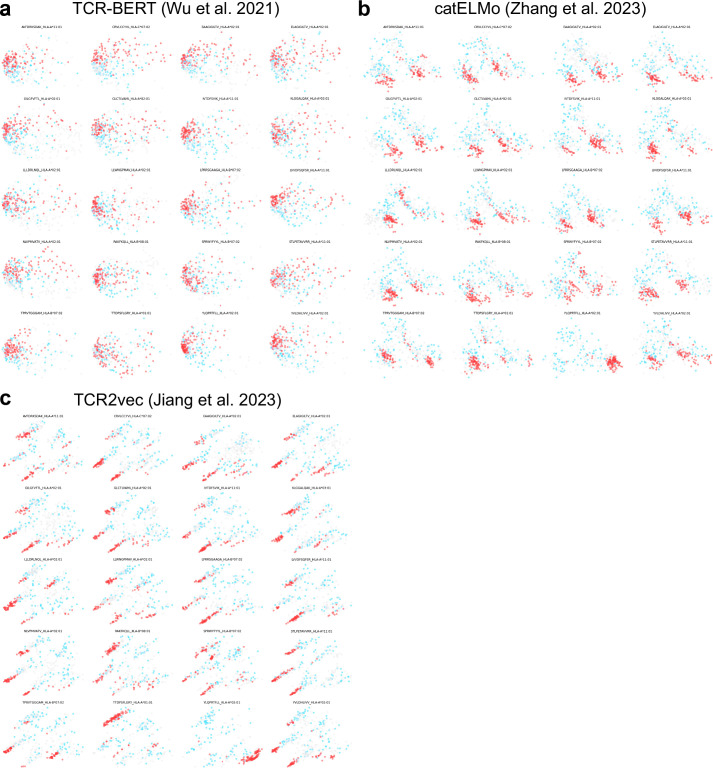
TCRT5 Metrics @1000. (**a**) Repertoire-level features of reference (validation target sequences) and generated CDR3*β*s. (**b**) Sequence logo plot generated from TCRT5 for the canonical GILGFVFTL (Influenza A), KLGGALQAK (CMV), and YLQPRTFLL (SARS-CoV2) from 1000 generations instead of 100. (**b**) TCRT5@1000 with beam search preferentially samples sequences at the right tail of OLGA generation probabilities. (**c**) Bar plots for individual pMHCs are overlaid on one another. (**d**) K-mer spectrum shift plot showing the Jensen-Shannon divergence between generated and reference sequences for TCRT5@1000. Error bars mark the mean and 1-standard deviation across validation pMHCs (*n* = 20). Mean soNNia values are shown per simulated run, with 1000 generations per pMHC per run over 100 simulations. (**e**) Heat map of Jaccard Index scores showing the generated sequence co-occurrence across different pMHC pairs at 1000 generations per pMHC. (**f**) Sankey diagram of TCRT5@1000 generations showing the validity as measured by nonzero generation probability, known binding status, and training set membership.

Finally, to test whether TCRT5 could generate known binders not seen during training, we analysed each generated sequence for biological validity, known specificity and training set membership of each of the translations and found that out of the 2,000 generations, 1,996 of them had non-zero generation probabilities, 181 were known binders and 7 were TCRs that were not seen during supervised training (Fig. 4g). In particular, one of these seven was not found in the pretraining set, indicating a real potential for sampling de novo TCRs. Moreover, they spanned multiple peptides, alleles and disease contexts: KLGGALQAK_A*03:01 (cytomegalovirus), LLWNGPMAV_A*02:01 (yellow fever virus), YLQPRTFLL_A*02:01 (SARS-CoV2) and YVLDHLIVV_A*02:01 (Epstein–Barr virus (EBV)), demonstrating that the performance was not localized to a single peptide or MHC. All analyses described above remained consistent when validated at a sampling depth of 1,000 sequences (Extended Data Fig. 6).

### TCRT5 achieves state-of-the-art performance on sparsely validated epitopes

**Fig. 5:**
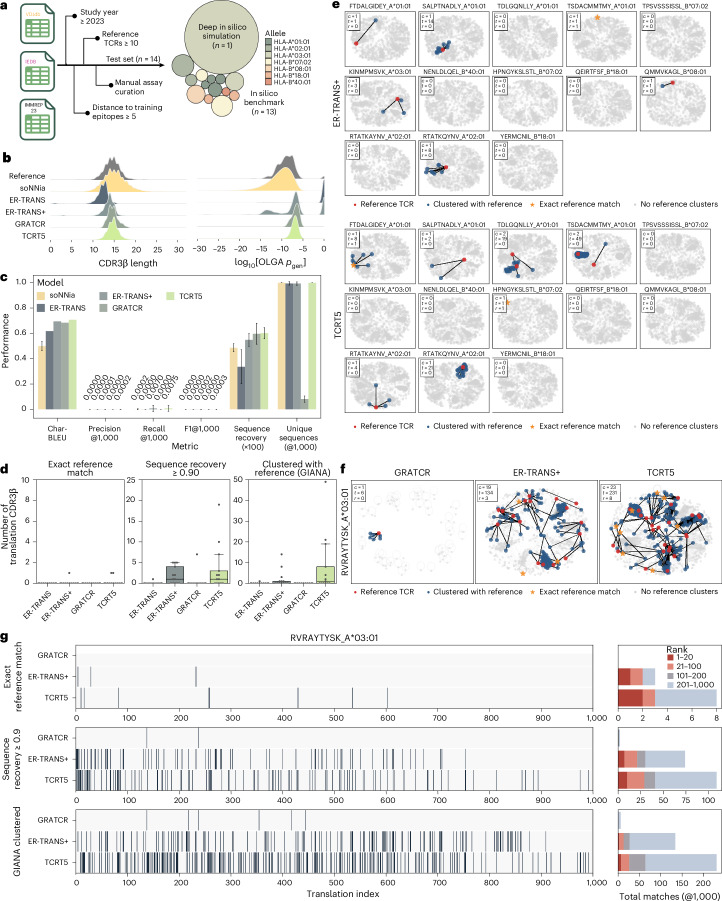
In silico benchmark on unseen epitopes. **a**, Schematic of the test set curation. Bubble plot of *n* = 14 test pMHCs coloured by allele, with the area corresponding to the number of unique cognate CDR3β sequences. The pMHC with the most reference TCRs was reserved for a deep in silico simulation and the remaining 13 were used for a sparse evaluation set. **b**, Repertoire-level features for reference CDR3β sequences and those generated by ER-TRANSFORMER (ER-TRANS), a modified ER-TRANSFORMER (ER-TRANS+), GRATCR and TCRT5 are shown as smoothed density curves. **c**, Benchmark metrics showing the aggregate performance of all models on the benchmark dataset (*n* = 13). soNNia-derived metrics are aggregated across pMHCs and 1,000 simulations to account for the stochasticity of generations. Error bars show mean ± s.d. **d**, Modified true-positive counts. Box and whisker plots showing the median and quartile values for benchmark pMHCs (*n* = 13) on exact matches, sequence recovery ≥ 90% and GIANA reference clustered. Whiskers extend to 1.5× the interquartile range. **e**, Network diagrams for model generations. Clusters with a known binder (red) and GIANA-clustered translations (blue) are highlighted. The number of highlighted clusters (*c*), the number of clustered translations (*t*) and the number of known binders sampled (*r*) are reported per pMHC. GRATCR is omitted due to zero reference-clustered sequences. **f**, Network diagrams for RVRAYTYSK (EBV). **g**, In silico simulation of RVRAYTYSK design challenge. Heat maps highlighting the rank of exact reference matches, sequence recovery ≥ 90% and GIANA reference-clustered sequences for each model are shown. For each metric, a summary bar plot counting the number of successes is shown, coloured by range. Panel **a** created with BioRender.com.

The goal of a conditional TCR generation model like TCRT5 is to sample TCRs against rare epitopes not seen during training, especially when few or no known TCRs exist. To better understand the utility of these models in such a real-world setting, we benchmarked against two publicly available models: ER-TRANSFORMER^17^ and GRATCR^18^. For a fair comparison, we curated a test set of high-confidence paired data from recent exports of VDJdb^27^, IEDB^28^ and the IMMREP2023 TCR-pMHC specificity competition^10^. We included studies after January 2023 with at least ten CDR3β sequences per epitope and a minimum edit distance (*D*_train_) of 5 to any training epitope (Fig. 5a). This resulted in 14 epitopes spanning seven HLA alleles. One EBV epitope, RVRAYTYSK (HLA-A*03:01), contained 895 unique CDR3β sequences and was reserved for a simulated in silico functional design assay. The remaining 13 were used to construct a sparse benchmark evaluation set.

To maximize the likelihood of exact sequence recovery, we sampled 1,000 sequences per model per pMHC. As a sanity check, we observed that all models produced sequences with comparable lengths and OLGA-derived *p*_gen_ distributions, further suggesting that beam search decoding favours common high-probability motifs irrespective of the model (Fig. 5b and Supplementary Note A.8). Since ER-TRANSFORMER frequently omitted the canonical N-terminal cysteine and C-terminal phenylalanine, we defined an ER-TRANSFORMER+ variant that appends these residues when missing, recovering more realistic sequences. Across the 13 sparse epitopes, although all conditional models outperformed an unconditional soNNia baseline, TCRT5 achieved the highest overall performance, even recovering an exact sequence match for FTDALGIDEY (A*01:01) and HPNGYKSLSTL (B*07:02) (Fig. 5c).

Given the rarity of exact matches and the functional relevance of sequence similarity, we next evaluated whether TCRT5-generated sequences were predicted to be functionally similar to known binders. We leveraged GIANA^29^, an unsupervised clustering model that demonstrated high cluster purity in a recent benchmark of TCR clustering methods^30^ as well as computed the number of generated sequences with ≥90% sequence identity to known binders—a threshold we found empirically to align with improved precision–recall metrics (Supplementary Note A.9). We found that TCRT5 generated more sequences with greater than 90% sequence identity as well as those that clustered with the reference sequences, with ER-TRANSFORMER+ performing comparably (Fig. 5d,e).

Finally, to assess whether TCRT5 prioritized functional sequences at higher ranks, we simulated a prospective screen by generating 1,000 sequences for RVRAYTYSK and compared them against the 895 known CDR3β sequences. Using GIANA to cluster the model outputs with known binders, we found that GRATCR, ER-TRANSFORMER+ and TCRT5 generated 6, 133 and 231 clustered sequences, respectively, corresponding to 1, 19 and 23 unique reference CDR3β sequences (Fig. 5f). Remarkably, TCRT5 also recovered eight known binders, compared with three for ER-TRANSFORMER+ and zero for GRATCR. Moreover, TCRT5 consistently generated more sequences in top-ranked positions, outperforming all baselines across all rank cut-offs (Fig. 5g). These results demonstrate that TCRT5 samples realistic TCR sequences and prioritizes functional candidates, highlighting its potential utility in real-world TCR generation scenarios.

### TCRT5 validates in vitro

**Fig. 6:**
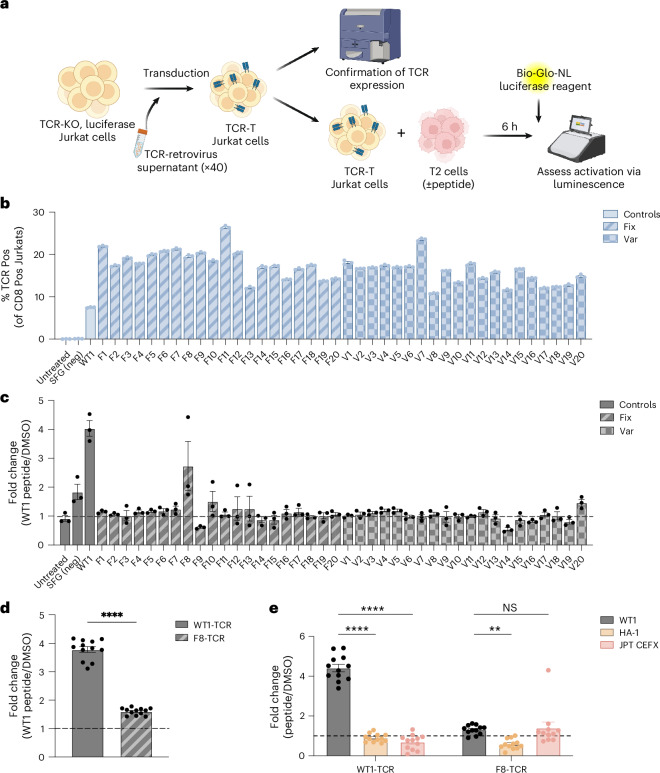
In vitro validation of TCRT5-generated CDR3β sequences. **a**, Schematic depicting the generation and functional validation of NFAT-luciferase reporter Jurkat cells containing the predicted CDR3β sequences. **b**, Expression of TCRαβ on CD8^+^ Jurkat cells following retroviral transduction (*n* = 3 technical replicates). **c**, Fold change of RLUs for the engineered Jurkat cells co-cultured with WT1 peptide-pulsed versus DMSO control-treated T2 cells (*n* = 3 technical replicates). **d**, Follow-up functional validation assay of RLU fold change for WT1 and F8 Jurkat cells (*n* = 12 technical replicates). **e**, Specificity assay. Fold change of RLUs for WT1 and F8 Jurkat cells following co-culture with WT1 peptide, minor histocompatibility antigen HA-1 or CEFX Ultra SuperStim versus DMSO-treated T2 cells (*n* = 12 technical replicates). All tests of significance were done using the Student’s *t*-test. Error bars for biological samples in **b**–**e** show the standard error of the mean. Var, variable; NS, not significant. ****P ≤ .0001, **P ≤ .01. Panel **a** created with BioRender.com. Source data

**Extended Data Fig. 7:**
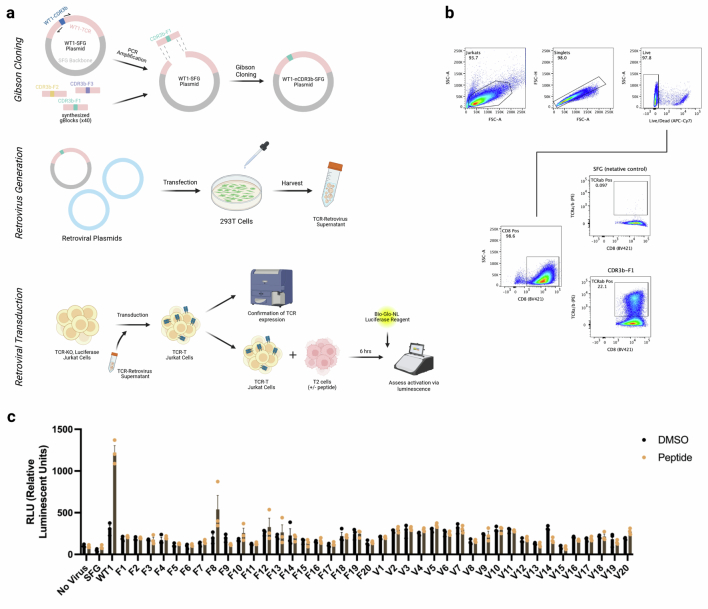
Supporting details for in vitro validation of predicted CDR3*β* sequences. (**a**) Detailed schematic of Gibson cloning generated CDR3*β* sequences into a retroviral expression plasmid containing a WT1 TCR sequence, retrovirus generation using 293T cells, and retroviral transduction to generate TCR-Jurkat cell lines for validation studies. (**b**) Gating strategy used to assess TCR expression. (**c**) Raw relative luminescence units (RLUs) for 40 generated CDR3*β* sequences stimulated with WT1 peptide or DMSO (*n* = 3 technical replicates). Error bars show SEM. Panel ‘**a**’ created in BioRender.

Next, we sought to experimentally validate TCRT5 generations against a non-viral epitope, a notable challenge given our training set composition. We selected an HLA-A*02:01 epitope derived from leukaemia-associated Wilms’ tumour antigen-1 (WT1; sequence: VLDFAPPGA; *D*_train_ = 4)^31^, a target with a strong positive control in an existing TCR-T^32^. To test TCR functionality from the generated CDR3β sequences, we swapped the CDR3β of the TCR-T (henceforth referred to as the WT1 TCR) with TCRT5-generated sequences. These TCR constructs were then expressed in a TCR-KO Jurkat cell line with a nuclear factor of activated T cells (NFAT) promoter upstream of the luciferase enzyme, a setup that enabled the rapid functional read-out of T cell activation via luminescence (Fig. 6a, Extended Data Fig. 7a,b and Supplementary Note A.10). To account for CDR3β length differences disrupting TCR folding, we tested two sequence sets: variable-length sequences (20 sequences sampled uniformly from 100 generations) and fixed-length sequences (the first 20 sequences with the native WT1 CDR3β length).

Surprisingly, all of the designed CDR3β-swapped gene sequences showed structurally viable TCR expression on the cell surface as assessed by flow cytometry (Fig. 6b), highlighting the feasibility of CDR3β grafting. Of the 40 engineered TCR constructs tested, one generated sequence, F8 (CASSVGLYNEQFF) from the fixed-length set, demonstrated a substantial increase in luciferase expression over the dimethyl sulfoxide (DMSO) controls (Fig. 6c,d and Extended Data Fig. 7c). In particular, this sequence was in the pretraining corpus but absent from our fine-tuned data, indicating that TCRT5 generated a naturally occurring TCR, correctly identifying it from vast unlabelled repertoires. Although F8’s activity was lower than the established WT1 TCR positive control, it demonstrated that TCRT5 can generate sequences capable of mediating epitope-specific functional activation. To assess F8’s specificity, we tested for off-target reactivity against two controls: the related HA-1 antigen (A*02:01, VLHDDLLEA) and CEFX Ultra SuperStim (CEFX), a highly immunogenic cocktail of 80 bacteria- and virus-derived MHC class I peptides. The WT1 TCR responded only to its cognate peptide, whereas F8 did not react to HA-1 but showed similar activation levels for both WT1 and CEFX pools (Fig. 6e). Although these results demonstrate TCRT5’s ability to elicit a functional response to an out-of-distribution epitope, the identified F8 TCR’s reactivity against CEFX suggests a level of polyspecificity that highlights the need for further refinement in target selectivity and dataset construction for future work.

## Discussion

In silico identification of TCRs that precisely target arbitrary pMHCs remains one of the great outstanding challenges in computational immunology^9^, requiring models to navigate a complex interaction network of cross-reactive TCR-pMHC specificities in the face of sparsely labelled data. Here we present TCR-TRANSLATE, a seq2seq framework adapting low-resource machine translation techniques to conditional CDR3β sequence generation, demonstrating the rapid sampling of antigen-specific repertoires in this data-sparse domain. Our systematic exploration reveals key insights about bespoke training methods, generation diversity and TCR polyspecificity, culminating in the generation of a TCR construct demonstrating functional activity against a therapeutically relevant target without post hoc optimization.

Interestingly, we found that pretraining had opposite effects on TCRT5 and TCRBART, probably due to the former’s span masking strategy being better suited to learn on CDR3β sequences given their short lengths and the standard masking rate of 15% (ref. ^33^). Although span masking forces models to understand higher-order *k*-mer motifs, token masking at 15% would mask two amino acids on average, providing minimal learning signal per epoch. Beyond these architectural findings, we observed the models’ tendency to preferentially sample polyspecific TCRs, especially in the bidirectional and multitask training regimes. This consistent trend suggests that the alignment of sequence spaces through self-consistency training may inadvertently prioritize empirically de-risked sequences or high-likelihood sequences that satisfy many input conditions, even at the cost of diversity.

Despite these inherent biases, our flagship TCRT5 model outperformed both ER-TRANSFORMER^17^ and GRATCR^18^ across all metrics on held-out epitopes. Interestingly, all models sampled sequences with high V(D)J generation probabilities via beam search, suggesting potentially limited generalizability to rare targets. This pattern is well documented in natural language, where beam search and the broader class of mode-seeking decoding algorithms are known to sample simpler subsequences^34^. In the TCR space, this creates an interesting paradox. Although ancestral sampling produced distributions more closely resembling the real cognate repertoire, beam search consistently outperformed it on F1 metrics^16^. This suggests a potential confirmation bias in our evaluation, where higher *p*_gen_ sequences may be over-represented in the reference set because they are more likely to be experimentally observed, effectively rewarding models for reproducing sampling biases rather than capturing true functional diversity.

The experimental validation of the F8 TCR construct represents the successful de novo design of a functional CDR3β, not seen during training, against a therapeutically relevant non-viral target. Remarkably, all 40 generated sequences showed viable surface expression, demonstrating the feasibility of CDR3β grafting. Although the overall hit rate (1/40) is low, it represents orders-of-magnitude improvement over traditional discovery methods. In particular, the F8 sequence was absent from our training data but exists in the unannotated form in iReceptor^35^, indicating that TCRT5 identified a naturally occurring TCR with previously unknown specificity. The F8 TCR’s cross-reactivity with the CEFX peptide pool but not with HA-1 suggests a level of learned polyspecificity that reflects both biological reality and limitations in our training data. This behaviour reveals a fundamental tension between therapeutic utility and the underlying biology. Although polyspecificity is an evolved feature that enables broad immune surveillance, its utility in clinical applications is diminished, creating a scenario in which natural TCR properties may conflict with therapeutic requirements.

The current iteration of our study has many limitations stemming from both our approach and an innate scarcity of available data. In the data-scarce regime, we were most limited in silico by our ability to evaluate the models. Metrics based on exact sequence recovery, sequence similarity thresholds (≥90%) and clustering serve as imperfect proxies for functional specificity, providing noisy estimates of performance. Functionally, our focus on the CDR3β loop requires a scaffold TCR, neglecting the crucial contributions of the α chain and V/J genes to antigen specificity^36,37^. Furthermore, all sampled sequences were validated without downstream prioritization, a design choice that preserved the unbiased evaluation of TCRT5, which does not fully reflect how a generative model would be used in a therapeutic discovery pipeline. Additionally, our choice of TCRT5-FT as the flagship model and the behavioural analyses we report were based on the validation set performance. However, we argue for its necessity given the severe data sparsity to evaluate pMHCs across multiple disease contexts. Importantly, TCRT5 demonstrates consistent, monotonic improvement in performance across training checkpoints, suggesting that our final model demonstrates real learning rather than random fluctuations to its parameters.

Experimentally, our validation was limited to a single non-viral epitope in a Jurkat NFAT-luciferase system, providing a useful but limited view of TCR-mediated T cell activation in the nucleus. This assay stops short of capturing the final read-out of cytotoxicity and cytokine secretion and simultaneously does not capture high-affinity, low-activity TCRs. Although we successfully validated a TCR against WT1, our modest hit rate (fixed length, 1/20; total, 1/40) and observed cross-reactivity with the CEFX peptide pool show the model’s current limitations in sampling fine-grained specificity signals, highlighting the need for richer, non-viral data. A broader experimental sweep to include diverse epitopes, running multimer stains and cytotoxicity screens to quantify upstream and downstream biological activities, and training on newer architectures and better datasets would help generalizability before expression in primary T cells.

Contrary to traditional TCR discovery processes, which have low yield, models like TCRT5 enable rapid hypothesis generation for rare epitopes with few to no known TCRs, drastically reducing the search space of possible cognate sequences. Through benchmarking on held-out epitopes, we showed that TCRT5 outperforms available conditional generation models across all reported metrics, including exact matches, sequence clustering and prioritization of known binders at higher ranks. More importantly, we validated this performance experimentally by identifying a functional TCR (F8) against an out-of-distribution, non-viral epitope without post hoc filtering, underscoring the model’s potential for out-of-the-box real-world utility. Although challenges remain, this work represents an important step towards achieving computationally guided targeting of arbitrary peptide sequences at will. As more data become available, the performance of generative models like TCRT5 is expected to improve, moving the field closer to scalable, high-precision TCR design for personalized immunotherapies that can rapidly respond to emerging threats and individual patient needs.

## Methods

### Sequence representation

We adopt the same seq2seq framework introduced in ref. ^16^, relaxing the direction of the pMHC→TCR source–target pairs to train on pMHC→TCR and TCR→pMHC, but evaluate on the former. To represent the TCR-pMHC trimeric complex, comprising three subinteractions (TCR-peptide, TCR-MHC and peptide-MHC) as a source–target sequence pair, we made a few simplifying assumptions that allowed for a more straightforward problem formulation. First, we assume a stable pMHC complex, reducing the problem space to a dimeric interaction between TCR and pMHC. Second, we focus on the variable amino acid residues at the binding interface. For the TCR, we use the CDR3β loop, a contiguous span of 8–20 amino acids that typically make the most contact with the peptide^38^. Similarly, for the pMHC, we use the whole peptide and the MHC pseudo-sequence, defined in ref. ^39^ as a reduced, non-contiguous, string containing the polymorphic amino acids within 4.0 Å of the peptide. We opt for a single-character amino-acid-level tokenization, primarily for its interpretability^40^. In addition to the 20 canonical amino acids, we use standard special tokens to encode semantic information pertaining to the structure of the sequences including the start of sequence [SOS], end of sequence [EOS], masking [MASK], padding [PAD] and a separator token [SEP] to delineate the boundary between the concatenated peptide and pseudo-sequence. For TCRT5, we additionally employ the use of sequence-type tokens [TCR] and [PMHC], retained from T5’s use of task prefixes^20^, to designate the translation direction:

TCRBART*:*

[SOS]EPITOPE[SEP]PSEUDOSEQUENCE[EOS]↔[SOS]CDR3BSEQ[EOS]

TCRT5*:*

[PMHC]EPITOPE[SEP]PSEUDOSEQUENCE[EOS]↔[TCR]CDR3BSEQ[EOS]

Of note, this formulation is extensible to other sequence representations of both TCR and pMHC by using the [SEP] token to delineate the α- and β-chain information for CDR3, multiple CDRs, and even full-chain sequence representations. Similarly, this approach can be used for the full MHC sequence as well.

### Dataset construction

#### Core parallel corpus

Our parallel corpus comprised experimentally validated immunogenic TCR-pMHC pairs taken from publicly available databases (McPAS^41^, VDJdb^27^ and IEDB^28^). All data were collected before May 2023. Additionally, we used a large sample of partially labelled data derived from the MIRA^42^ dataset, which contained CDR3β and peptide sequences, but contained MHC information at the haplotype resolution instead of the actual presenting MHC allele. Therefore, the presenting MHC allele was inferred from the individual’s haplotype using MHCflurry 2.0’s^43^ top-ranked presentation score for the listed alleles. Of importance, these allele-imputed examples were not used in the evaluation. To aggregate the data spanning various sources, formats and nomenclature, we mapped the columns from each individual dataset to a common consensus schema and concatenated the data along the consensus columns. Missing values were reasonably imputed based on other information for that data instance. To keep only the cytotoxic (CD8^+^) T cells, we filtered the instances in which the cell type was provided or the HLA allele was of MHC class I. Once the data were aggregated and the values were imputed, we applied the following column-level standardization for each source of information:

- CDR3β, epitope and MHC pseudo-sequence: all amino acid representations were normalized using the ‘tidytcells.aa.standardise’ function found in the tidytcells Python package^44^.
- TR genes: the tidytcells package^44^ was once again used to standardize the nomenclature surrounding the TCR genes (for example, TRB-V and TRB-J).
- HLA allele: HLA allele information was parsed and standardized to the HLA-[A,B,C]*XX:YY format using the ‘mhcgnomes’ package (https://github.com/pirl-unc/mhcgnomes), and only the parsed entities that identified as alleles were retained whereas those with serotype and class-level resolution were filtered. For a small number of cases in which mhcgnomes identified an allele group but was unable to find/parse protein-level information, we imputed the protein field by incrementing from ‘*01’ until a matching IMGT allele was found. Although this step has the potential of introducing differences between the imputed pseudo-sequence and the ground truth, we anticipate this source of noise to have a minor effect as the MHC pseudo-sequence is well conserved within the serotype. HLA alleles were imputed when necessary and then normalized using the mhcgnomes package to the standard HLA-[A,B,C]*XX:YY format.

Once aggregated, only entries derived from human studies with MHC class I peptides were retained. Additionally, entries with the minimal information of HLA, peptide and CDR3β were retained. No other data filtration was performed for the training and validation splits.

#### Training/validation split

To assess the feasibility of having the models sample antigen-specific sequences for unseen epitopes, we held out a validation set of the top-20 most-target-rich pMHCs. We trained on the remaining data, further removing the occurrences of the held-out, epitope-bound alternate MHCs to ensure a clean validation split (Fig. 1c). We retained training sequences with a low edit distance to the validation pMHCs to better understand their influence on performance. The degree to which these sequences exhibit training set similarity is reflected in Extended Data Table 1. The parallel corpus was subsequently de-duplicated to remove near duplicates (peptides with the same allele and a ≥6-mer overlap), which we found to marginally help the overall performance, in accordance with ref. ^45^. This resulted in a final dataset split of ~330k training sequence pairs (*N* = 6,989 pMHCs) and 68k validation sequence pairs (*N* = 20 pMHCs). A key limitation of our validation dataset is its bias towards mainly viral epitopes and a very narrow HLA distribution towards well-studied alleles.

#### Unlabelled ‘monolingual’ data

We hypothesized that pretraining the encoder–decoder model using self-supervised methods on pMHC and TCR sequences could help boost the translation performance of the model by learning better representations for source and target sequences, as that in ref. ^46^, which crucially has been shown to improve performance in the low-resource setting^21^. For the unlabelled pMHC sequences, we used the positive MHC ligand binding assay data from IEDB (*N* ≈ 740k)^28^. For the TCR sequences, we used around (*N* ≈ 14M) sequences from TCRdb^47^, out of which around 7M CDR3β sequences were unique. For this dataset, we chose to retain duplicate CDR3β sequences as the TCRdb was amassed over multiple studies and populations; therefore, we felt that the inclusion of duplicate CDR3β sequences was reflective of convergent evolution in the true unconditional TCR distribution.

#### Benchmark ‘test’ data

To fairly compare TCRT5 against external models ER-TRANSFORMER^17^ and GRATCR^18^, we looked for data that would not advantage any one model over the other. This meant that we needed to find data that were not from any training set or validation set, which would have introduced leakage via model selection. Since GRATCR was fine-tuned exclusively on MIRA data, filtering for our training and validation sets would cover the GRATCR model. However, since we were not able to find the training set for ER-TRANSFORMER, we adopted a slightly more stringent data inclusion policy. To account for both ours and ER-TRANSFORMER’s dataset, we aimed to find paired TCR-pMHC data from recent studies (2023 onwards) and filter for epitopes that were at least five amino acid edits away from anything in our training set. For its distributed use and well-characterized performance, the IMMREP2023 TCR specificity competition^10^ was used along with recent exports from VDJdb and IEDB, which were accessed on 25 March 25 and 1 April 2025, respectively. To ensure that quality examples were taken from VDJdb, entries with a confidence score of ≥2 were chosen. After applying our filtering criteria, we were left with four pMHCs from the IMMREP2023 dataset, four pMHCs from IEDB and eight pMHCs from VDJdb. After manually examining the 16 pMHCs and validating their assay conditions, two pMHCs from VDJdb that shared the same peptide ‘RPIIRPATL’ were dropped due to their inclusion in a 2021 study. The final test set consisted of 14 epitopes with the ‘RVRAYTYSK’ epitope, which contained 895 unique CDR3β sequences, being removed from the benchmark set to have *n* = 13 pMHCs for the benchmark and the ‘RVRAYTYSK’ epitope as an in silico simulation. The degree to which these sequences exhibit training set similarity is reflected in Extended Data Table 2.

Supplementary Note A.11 provides more information.

### Model training

#### Pretraining

TCRBART was pretrained using masked amino acid modelling (BERT style^48^), whereas TCRT5 utilized masked span reconstruction, learning to fill in randomly dropped spans with lengths between 1 and 3. Of importance, neither model was trained on complete sequence reconstruction to reduce the possibility of memorization during pretraining. Both models were trained on unlabelled CDR3β and peptide-pseudo-sequences, simultaneously pretraining the encoder and decoder, inspired by the MASS/XLM approach^49,50^. Unlike MASS/XLM, we omitted per-token learned language embeddings, allowing TCRBART to learn from the size differences between CDR3β and pMHC sequences and TCRT5 to use the [TCR] and [PMHC] starting tokens. To address the imbalance in sequence types, we upsampled sequences for a 70/30 TCR/pMHC split.

#### Direct training/fine-tuning

For the parallel data, we used the same three training protocols (baseline, bidirectional and multitask) for direct training from random initialization as well as fine-tuning from a pretrained model. This was done by extending the standard categorical cross-entropy loss function (equation (1)), favoured in seq2seq tasks for its desired effect of maximizing the conditional likelihoods over target sequences^51,52^. For the baseline training, we used the canonical form of the cross-entropy loss, as shown below:

1 $$\begin{array}{rcl}{\mathcal{L}}&=&{\rm{CE}}({\bf{y}},\hat{{\bf{y}}})=-\mathop{\sum }\limits_{i=1}^{n}{{\bf{y}}}_{i}\log [{\hat{{\bf{y}}}}_{i}]\\ &=&-\mathop{\sum }\limits_{i=1}^{n}\mathop{\sum }\limits_{j-1}^{k}{y}_{ij}\log [{p}_{\theta }({y}_{ij}| {\bf{x}})]\end{array}.$$

The bidirectional and multitask models were trained using multiterm objectives, forming a linear combination of individual loss terms corresponding to the cross-entropy loss of each task/direction.

2 $${{\mathcal{L}}}_{\rm{bidxn}}={{\mathcal{L}}}_{pmhc\to tcr}+{{\mathcal{L}}}_{tcr\to pmhc}$$

3 $${{\mathcal{L}}}_{\rm{multi}}={{\mathcal{L}}}_{mlm}+{{\mathcal{L}}}_{pmhc\to tcr}+{{\mathcal{L}}}_{tcr\to pmhc}$$

To mitigate the effects of model forgetting with stacking single-task training epochs, we shuffled the tasks across the epoch using a simple batch processing algorithm (Algorithm 1). After the batch was sampled, it was rearranged into one of four seq2seq mapping possibilities and trained on target reconstruction with the standard cross-entropy loss, which was used for backpropagation. In this way, we could ensure that the model was simultaneously learning multiple tasks during training. For the bidirectional model, this was straightforward as we could swap the input and output tensors during training to get the individual loss contributions of ${{\mathcal{L}}}_{\rm{pmhc\to tcr}}\,{\text{and}}\,{{\mathcal{L}}}_{tcr\to pmhc}$ (equation (2)). For the multitask model, the mapping possibilities are (1) pMHC→TCR, (2) TCR→pMHC, (3) masked/corrupted pMHC*→pMHC and (4) masked/corrupted TCR*→TCR, which combine to form ${{\mathcal{L}}}_{\rm{multi}}$ (equation (3)). These tasks and sequence mappings as seen by TCRBART and TCRT5 are summarized in Fig. 2b.

##### Algorithm 1

Multitask training step.

Batched input: source pMHCs, **X**; target TCRs, **Y**

Sample *a* ≈ Bernoulli(0.5)

**if** *a* > 0.5 **then**

Swap **X** and **Y**

Compute attention masks

**end if**

Sample *b* ≈ Bernoulli(0.5)

**if** *b* > 0.5 **then**

Set **X** = X* and **Y** = **X**

Compute attention masks

**end if**

**do** Predict $\hat{{\bf{Y}}}=\phi ({\bf{X}})$ and gradient updates on CE(**y**, $\hat{{\bf{y}}}$)

For the purposes of comparison between models originating from different training schemes, each of the models was trained for 20 epochs, from which the checkpoint with the highest average overlap to the known TCR reference set (F1 score) was chosen. We chose this approach to characterize the models’ real-world potential under optimal conditions, as opposed to training for a fixed number of steps or even a fixed number of steps per task (Supplementary Note A.6).

### Evaluation

To evaluate antigen specificity, we build our framework around sampling exact CDR3β sequences from published experimental data on well-characterized validation epitopes not seen during training. This approach has an interpretable bias compared with black-box error profiles, at the cost of potentially under-representing actual performance. We calculate sequence-similarity-based metrics beyond exact overlap to create a more robust evaluation framework, and characterize their concordances for future use on epitopes with fewer known cognate sequences. Broadly, our metrics can be summarized as evaluating the accuracy of the returned sequences, their diversity or some combination of the two. They are summarized in brief below:

#### Accuracy metrics

- Char-BLEU: following BLEU-4 (ref. ^53^), the character-level BLEU calculates the weighted *n*-gram precision against the *k* = 20 closest reference sequences to abate the unintended penalization of accurate predictions under a large reference set. We use NLTK’s ‘sentence_bleu’ function to calculate a single translation’s BLEU score and the ‘corpus_bleu’ function to compute the BLEU score over an entire dataset.
- Native sequence recovery: we compute the index-matched sequence overlap with the closest known binder of the same sequence length, when available. This is the same as the length-normalized Hamming distance. The Levenshtein distance normalized to the length of closest reference was used for cases in which a size-matched reference did not exist.
- mAP: borrowed from information retrieval, mAP measures the average precision across the ranked model predictions. Here we rank the generations by model log-likelihood scores and take the average of the precisions at the top-1, top-2, top-3… top-*k* ranked outputs. Then, we take the mean over the various pMHCs’ average precision values to get the mAP. This metric gauges the accuracy of the model as well as the calibration of its sequence likelihoods.
- Biological likelihood: to assess the plausibility of model outputs independent of antigen specificity or labelled data, we compute the generation probability of predictions using OLGA, a domain-specific generative model that infers CDR3β sequence likelihood^26^.

#### Diversity metrics

- Total unique sequences: as a measure of global diversity, we compute the number of total unique generations across the top-20 validation pMHCs as a diversity metric that captures model degeneracy and input specificity. This metric is a function sampling depth and is dependent on the relatedness or model-perceived relatedness of the input epitopes in a dataset.
- Jaccard similarity/dissimilarity index: the Jaccard index or the Jaccard similarity score is used to measure the similarity of two sets and is calculated as the size of the intersection divided by the union of the two sets. Since the Jaccard index is inversely proportional to diversity, one minus the Jaccard index is used to represent diversity between two sets.
- Positional *Δ*entropy: to quantify the change in diversity between the models’ outputs and the reference distribution per CDR3β position, we report *H*(*q*_*i*_) – *H*(*p*_*i*_) over the Kullback–Leibler divergence to get a signed change in entropy between the amino acid usage distribution of reference distribution *q* and sample distribution *p* at position *i*.

#### Both

- Precision@*K*: borrowed from information retrieval, this metric is calculated by sampling *K* sequences from the model, with the key distinction that we do not include rank. Here we count the true positives as the exact sequence overlap to the reference target sequences and false positives are chosen, although restrictively, as sequences that do not occur in the reference set. These quantities are combined to compute precision as follows:
  $$\,\text{Precision}=\frac{\text{True Positives (TP)}}{\text{True Positives (TP)}+\text{False Positives (FP)}\,}.$$
- Recall@*K*: also taken from information retrieval, this metric uses exact sequence overlap to measure the model’s ability to sample the breadth of reference sequences, which we calculate to be the minimum between *K* and the number of total reference sequences to ensure this metric ranges from 0 to 1:
  $$\,\text{Recall}=\frac{\text{True Positives (TP)}}{\min(K,\text{Total Reference Sequences})}.$$
- F1@*K*: the F1 score is computed as the harmonic mean of precision and recall, useful for its ability to capture a balanced picture between precision and recall:
  $$\,\text{F1}=2\times \frac{\text{Precision}\times \text{Recall}}{\text{Precision}+\text{Recall}}.$$
- *k*-mer spectrum shift: as used in the DNA sequence design space^54^, the *k*-mer spectrum shift measures the Jensen–Shannon (JS) divergence between the *k*-mer usage frequency distributions of two sets of sequences across different values of *k*. Here we compare the JS divergence between the distribution of *k*-mers derived from a pMHC’s model generations and its reference set of sequences.

### TCRT5 data ablation

To evaluate the impact of specific training decisions, we conducted an ablation study by removing key complexities of our training and data pipelines and measuring their effects on model performance. We started with our chosen model, TCRT5, fine-tuned on the single-task TCR generation with semisynthetic MIRA^42^ data. Next, we retrained the model without the MIRA data for an equivalent number of steps to assess its contribution. Finally, we removed pretraining altogether, training a model on the reduced dataset from random initialization.

To avoid over-representing the performance of the model trained on MIRA data on similar validation examples, we specifically removed three pMHCs that were a single-edit distance from a MIRA example with a greater than 5% overlap in their cognate CDR3β sequences (LLLDRLNQL, TTDPSFLGRY and YLQPRTFLL) from the validation set. For all the models, we used the same checkpoint heuristic, selecting the model with the highest F1 score.

### In silico benchmark

#### GRATCR

For running GRATCR on the test set peptides, we followed the instructions provided by the GRATCR team^18^ (https://github.com/zhzhou23/GRATCR). We ran the beam search decoding as provided. Since conditional likelihoods were not output by their beam implementation, the sampled sequence index was used as the translation rank. The script to sample the fine-tuned GRATCR was used as follows:

python GRA.py –data_path="./data/benchmark_peptides.csv"

–tcr_vocab_path="./Data/vocab/total-beta.csv"

–pep_vocab_path="./Data/vocab/total-epitope.csv"

–model_path="./model/gra.pth" –bert_path="./model/bert_pretrain.pth"

–gpt_path="./model/gpt_pretrain.pth" –mode="generate"

–result_path="./gratcr_benchmark_results.csv" –batch_size=1 –beam=1000

#### ER-TRANSFORMER

ER-TRANSFORMER was run using the unique amino acid model for a more direct comparison to TCRT5. We utilize the seq_generate method as described in their codebase with the default parameters shown in https://github.com/TencentAILabHealthcare/ER-BERT/ under Code/evaluate_seq2seq_MIRA.py as used by the ER-BERT team^17^. The translation rank was computed in the same manner as for TCRT5 using the Hugging Face infrastructure around model.generate. The code for sampling the ER-TRANSFORMER is shown below:

def seq_generate(input_seq, max_length, input_tokenizer, target_tokenizer, beams, k=1000):

input_tokenized = input_tokenizer(" ".join(input_seq),

padding="max_length",

max_length=max_length,

truncation=True,

return_tensors="pt")

input_ids = input_tokenized.input_ids.to("cpu")

attention_mask = input_tokenized.attention_mask.to("cpu")

outputs = model.generate(input_ids,

attention_mask=attention_mask,

num_beams=beams,

num_return_sequences=k)

output_str = target_tokenizer.batch_decode(outputs, skip_special_tokens=True)

output_str_nospace = [s.replace(" ", "") for s in output_str]

output_str_nospace = [s for s in output_str_nospace if s != ""]

return output_str_nospace

Additionally, we observed that the ER-TRANSFORMER performance was greatly improved using a post hoc editing step to the translations by simply adding a leading cysteine and ending phenylalanine wherever missing. Although this decreased the number of unique sequences, indicating that ER-TRANSFORMER was sampling both sequences with and without the required C and F, we felt that the large increase in accuracy warranted its inclusion for a fair benchmark and annotate this amended model ER-TRANSFORMER+, which we hold as a fairer comparison of the methods.

#### Modified F1 scores

In the sparse setting, evaluating the model performance using exact sequence recovery is zero inflated when this may not be the case if sufficient known binders were available. To help alleviate this, we took a principled approach of calling sequences true positives. The first was using sequence recovery values of >90% to a known reference CDR3β. Second, we used the GIANA 4.1 (ref. ^29^) clustering algorithm to cluster the generated samples with known reference sequences. Purported positives were the generated samples that clustered with a reference sequence. GIANA was run using only CDR3β information and all of the default settings using the following command:


python GIANA4.1.py -f cdr3b_input_file_path -v False


### In vitro validation

To further evaluate the ability of TCRT5 to generate epitope-specific CDR3β sequences for sparsely validated epitopes, we attempted to experimentally characterize a list of predicted CDR3β sequences for leukaemia-associated antigen, the HLA-A*02:01 presented WT1 (VLDFAPPGA)^31^ to be grafted on a well-characterized TCR-T^32^ using the sequence identified in ref. ^55^. From the list of generated CDR3β sequences, we selected 40 for in vitro validation. We chose 20 sequences of the same length as the original CDR3β sequence (13 AA) by oversampling TCRT5 and choosing the first 20 sequences of length 13. Additionally, we chose 20 sequences of variable CDR3β lengths by sampling 100 sequences from TCRT5 and taking every fifth sequence starting from the first, ranging from 15–17 AA long.

#### Retroviral transduction

Predicted CDR3β sequences (Extended Data Table 3) were synthesized as gBlocks (IDT, custom) and cloned into a standard SFG retroviral backbone vector^56^ containing the full-length WT1 TCR sequence. Sequences were codon optimized for expression in human cells and cloned plasmids were validated by Oxford Nanopore sequencing (Plasmidsaurus). TCR-retroviral supernatants were generated using 293T cells and co-transfection of the TCR-SFG, RDF and PegPam3 plasmids with GeneJuice Transfection Reagent (Sigma, 70967-5). Viral supernatants were harvested at 48- and 72-h post-transfection, snap frozen and stored at –80 °C. Transductions were performed using RetroNectin (Takara, T100A) according to the manufacturer’s recommendations.

#### TCR expression

TCRs were transduced into a genetically engineered Jurkat cell line (Promega, GA1182). The cell line is deficient in endogenous α and β chains (TCR-KO) and constitutively expresses both CD4 and CD8 co-receptors. Additionally, the TCR-KO Jurkats are engineered to express an NFAT-inducible luciferase reporter construct. Following transduction, TCR expression on the cell surface was evaluated by flow cytometry. Before staining, cells were incubated with 50-nM dasatinib for 30 min at 37 °C, shown to improve T cell staining^57^. TCR-Jurkats were then labelled with the following fluorochrome-labelled monoclonal antibodies: CD8-BV421 (BioLegend, 344748) and TCRα/β-PE (IP-26, BioLegend, 984702). Samples were also stained for viability using Live/Dead Fixable Near-IR (Thermo, L10119) and run on a BD Fortessa flow cytometer (BD Biosciences). Analysis was performed with FlowJo (v. 10.10.0)

#### T cell activation and luminescence read-out

To assess T cell activation, 4 × 10^5^ TCR-T Jurkats were cultured in a 96-well plate for 6 h with peptide or DMSO-pulsed T2 cells at a 10:1 effector-to-target ratio. Before co-culture, T2 cells were pulsed overnight at 1 × 10^6^ cells ml^−1^ supplemented with 10-μM peptide. Peptides were synthesized at GenScript with >95% purity (GenScript, custom). Luciferase expression was measured using the Bio-Glo-NL assay system (Promega, J3081) according to the manufacturer’s protocol. Luminescence was measured as relative luminescent units (RLUs) using a BioTek Synergy 2 microplate reader. All the reported values were normalized by subtracting the average luminescence values of the media control wells. Comparisons against the peptide-null control (DMSO) are reported as fold change values. Selected TCRs were also screened against a set of control peptides: HA-1 (VLRDDLLEA), a minor histocompatibility antigen commonly targeted in leukaemia, and CEFX Ultra SuperStim Pool MHC-I Subset (JPT, PM-CEFX-4), a mix of 80 class I bacterial and virally derived peptides known to react across a range of class I MHC alleles.

### Statistics

Fisher’s exact test (one sided) was used to determine the *P* values for *P*_bidxn_ and *P*_multi_ for quantifying the difference in number of polyspecific TCRs sampled. This was computed using the ‘scipy.stats’ Python library. Pairwise Student’s *t*-test was computed for tests of significance between peptide and DMSO controls for all biological validation data.

## Supplementary information

Supplementary Information Supplementary Notes A1–A11.

## Source data

Source Data Fig. 6 Luminescence data for WT1 peptide-pulse activation assay.

## Figures and Tables

**Extended Data Table 1:**
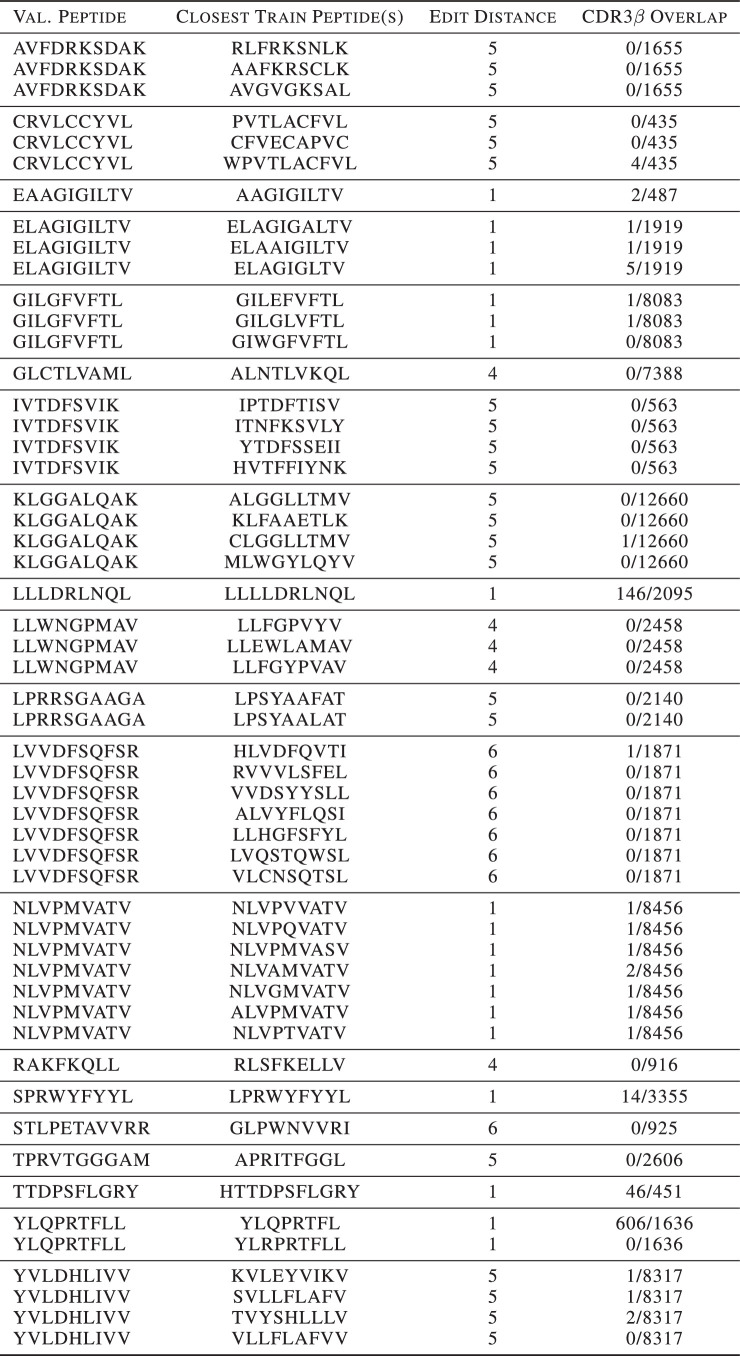
Characterization of train/validation overlap

.

**Extended Data Table 2:**
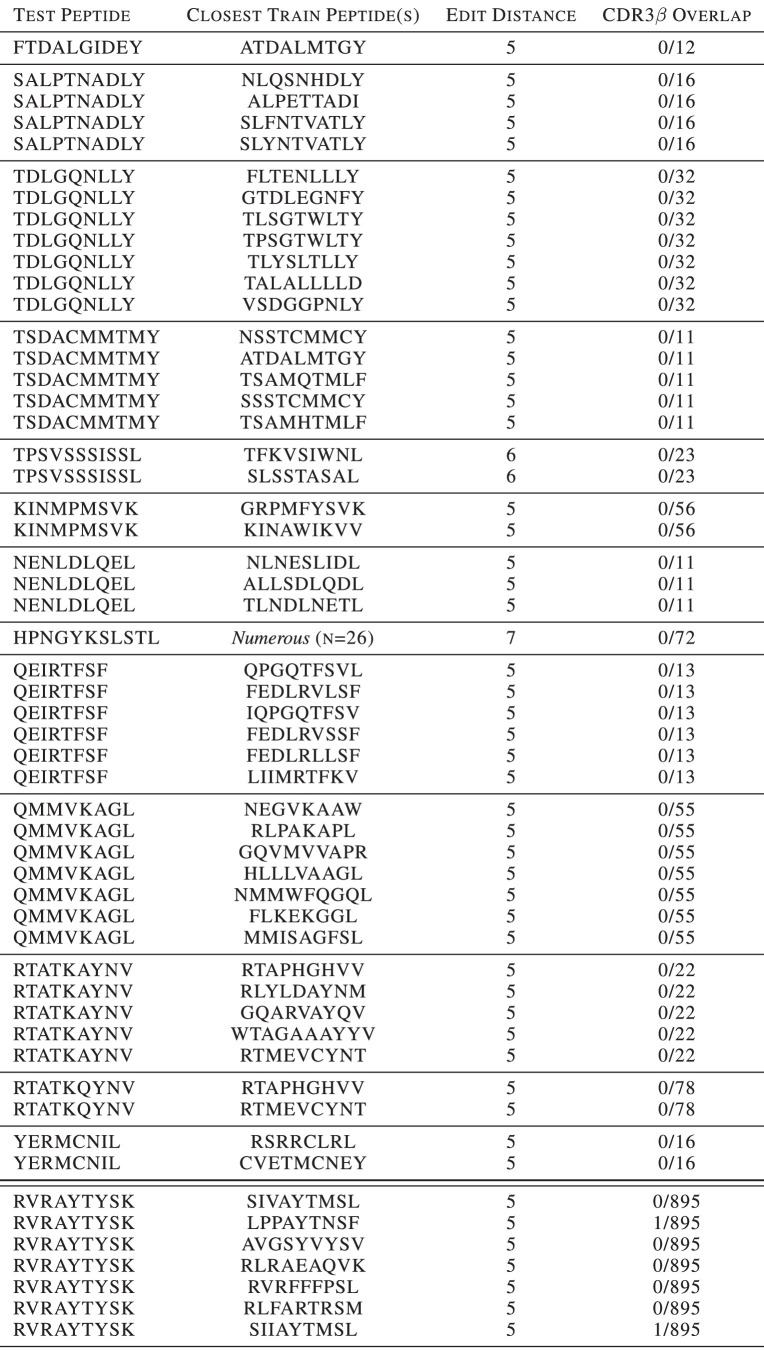
Characterization of train/test overlap

.

**Extended Data Table 3:**
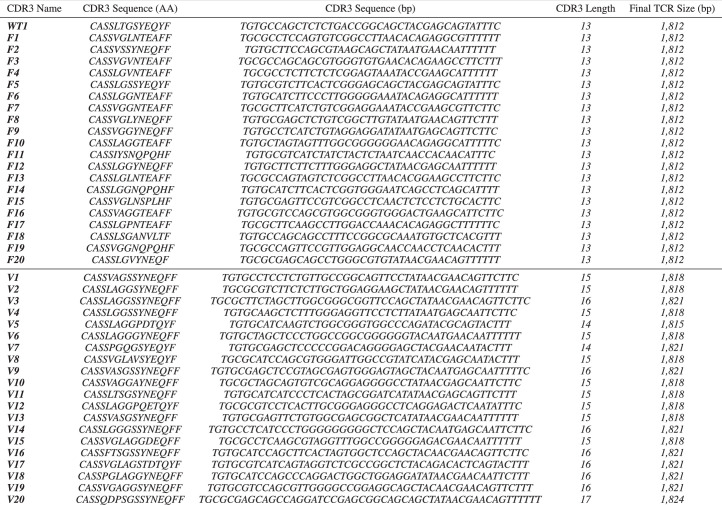
TCRT5 predicted sequences and associated constructs for Wilms’ Tumor Antigen 1 (WT1)

## Data Availability

All sequence data, generations and computational results used for the paper figures are available via GitHub at https://github.com/pirl-unc/tcr_translate. TCR and pMHC sequences are taken from publicly available databases (IEDB, McPAS, VDJdb and MIRA) and are provided in the source–target pair format used for seq2seq training. Wet-laboratory validation data including flow panel read-outs and luminescence plate reader output are available via Zenodo (10.5281/zenodo.15724161)^58^. Source data are provided with this paper.

## Code availability

All code used for training and evaluating TCRT5 is available via GitHub (https://github.com/pirl-unc/tcr_translate) or via Zenodo (10.5281/zenodo.15068617)^59^. For ease of use, the model and tokenizer for TCRT5 can be downloaded from Hugging Face at https://huggingface.co/dkarthikeyan1/tcrt5_ft_tcrdb. Additionally, the pretrained TCRT5 is available via Hugging Face at https://huggingface.co/dkarthikeyan1/tcrt5_pre_tcrdb.
