# Supplementary material for: Conditional generation of real antigen-specific T cell receptor sequences
Source: Nat Mach Intell. 2025 Sep 8;7(9):1494–509. doi: 10.1038/s42256-025-01096-6 (PMC12460172; doi:10.1038/s42256-025-01096-6)
Supplement: Supplementary file 1 — Supplementary Notes A1–A11. [file 42256_2025_1096_MOESM1_ESM.pdf]

nature machine intelligence

Supplementary information https://doi.org/10.1038/s42256-025-01096-6

Conditional generation of real antigen-specific T cell receptor sequences

In the format provided by the authors and unedited

Contents

A Supplementary Notes **2**

A.1 Model Architecture . . . 2

A.2 Quantitative Breakdown of Data Constraints on Model Performance . . . 2

A.3 Choice of Decoding Algorithm . . . 5

A.4 Hyperparameter Optimization . . . 5

A.5 Evaluation Methodology . . . 7

A.6 Checkpoint Selection . . . 7

A.7 TCRT5 Data Ablation . . . 9

A.8 Revisiting Beam search for CDR3β sequences . . . 9

A.9 Metric Correlation . . . 10

A.10 Functional Validation of Computationally Designed TCR Sequences . . . 12

A.11 Dataset Composition . . . 12

A Supplementary Notes

A.1 Model Architecture

We choose BART [1] and T5 [2], both encoder:decoder transformer models, for the model class's demonstrated performance on robust benchmarks spanning various seq2seq tasks in the broad NLP setting [3, 4]. Compared to the encoder-only transformers like BERT [5], which are great at learning rich feature representations of sequences, and decoder-only models such as GPT[6] that are adept at generating coherent text from prompts, the encoder-decoder architecture combines learning rich latent representations of input sequences and sampling meaningful target sequences in a robust manner. This is especially useful when the mapping between source and target sequences between different lengths, different languages, or otherwise complicated [7]. Both TCRBART and TCRT5 are thus specific implementations of bidirectional encoders coupled with autoregressive decoders, whose transformer-block architectures do not diverge from their inspirations (Additional details may be found in the original papers). Given the many-to-many mapping of the problem and somewhat varying lengths between source-target sequences, we leverage the encoder:decoder framework to learn rich representations of the pMHC which is used to sample diverse and accurate CDR3β sequences by the decoder. There are a few differences between the implementations of the BART and T5 models. While the BART model follows the implementation of the original encoder:decoder transformer introduced in [8], T5 makes a few notable changes (Main Figure 1b). The main differences are in the implementation of the layer norm (T5 implements the root mean squared layer-norm and does away with a linear bias), the location of the layer norm (T5 opts for layer-norm prior to the attention mechanism), the encoding of positional information (T5 uses a linear attention bias to encode relative positional information), and dropout (T5 uses dropout gratuitously across the flow of information through the model). The intuition/effects of these changes can be found in the original T5 paper [2].

A.2 Quantitative Breakdown of Data Constraints on Model Performance

For recall-based metrics, nonzero seq2seq performance is predicated on a sufficiently representative number of reference target sequences. Given that we are evaluating generations where even the most represented pMHC has on the order of $\approx$ 10,000 observed sequences out of a theoretical max of 10^6 [9, 10] (1% of the total diversity), we are in a regime where observed model performance is on the lower end of theoretical performance. This is because in practice, we are evaluating the model not only on its ability to generate correct target sequences, but are inadvertently asking it to generate target sequences that resemble those that have been experimentally validated, not necessarily related to functional veracity (Figure S1a). We distill this intuition into a probabilistic framework to contextualize the limits on recall-based metrics (i.e. F1 score) for model evaluation, given the amount of data that currently exists:

Assume we have a held-out pMHC ($pMHC_i$) with a theoretical set C of cognate CDR3β sequences, of which a subset of sequences have been experimentally validated (observed). We can model the likelihood of the generated sequences belonging to the observed set using a composite distribution linking the binomial and hypergeometric distributions. Given a model that samples n CDR3β sequences conditioned on that pMHC, we can define Z to be an unobservable binomially distributed random variable representing the number of correct (but not necessarily observed) generated CDR3β sequences.

$$\Pr(Z = z; \theta_i) = \binom{n}{z} \theta_i^z (1 - \theta_i)^{n-z}$$

where: Z = Random Variable: unobservable number of correct sequences that are in the reference set
n = Number of generated translations
θ_i = True model accuracy for a given $pMHC_i$

Then we can construct a conditional distribution of number of correct and observed sequences $Y|Z$ according to:

$$\Pr(Y = y|Z = z) = \frac{\binom{K}{y}\binom{N-K}{z-y}}{\binom{N}{z}}$$

where: Y = Random Variable: observed number of correct sequences that have been experimentally validated.
N = Number of total cognate sequences (ground truth, partially observed)
K = Number of experimentally validated cognate sequences
n = Number of generated sequences
z = Sample size (number of correct generated sequences, observed through Y)

This gives a joint distribution:

$$\begin{aligned}\text{Pr}(Y = y, Z = z) &= Pr(Y = y|Z = z)Pr(Z = z) \\ &= \frac{\binom{K}{y}\binom{N-K}{z-y}}{\binom{N}{y}}\binom{n}{z}\theta_i^z(1-\theta_i)^{n-z}\end{aligned} \tag{1}$$

By marginalizing on Y we get the following equation, whose PMF and expectation we plot in Figure S1b-c:

$$\begin{aligned}\text{Pr}(Y = y) &= \sum_{z}^{n} Pr(Y = y|Z = z)Pr(Z = z) \\ \text{Pr}(Y = y; N, K, n, \theta_i) &= \sum_{z=y}^{n} \frac{\binom{K}{y}\binom{N-K}{z-y}}{\binom{N}{y}}\binom{n}{z}\theta_i^z(1-\theta_i)^{n-z}\end{aligned} \tag{2}$$

We posit that this framework may be useful in characterizing model performance via estimating the parameter θ_i for pMHCs using Bayesian methods or jointly estimating Z_i and θ_i through the Expectation Maximization algorithm. We leave this for future exploration and use the above for contextualizing evaluation performance in the current data regime.

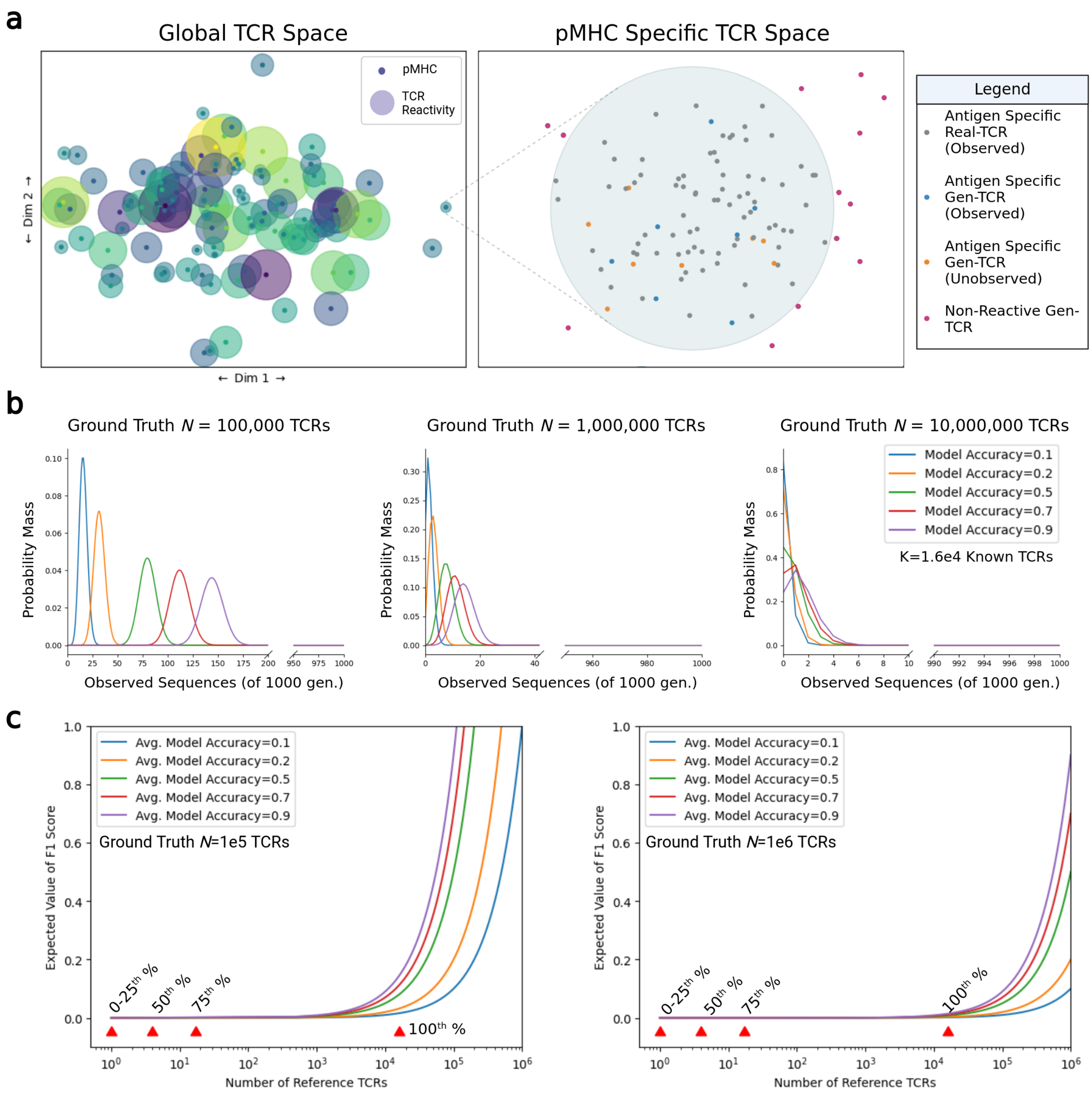

Supplementary Figure 1: Characterization of the TCR:pMHC Data Landscape. (a) Illustrative diagram showing an idealized rendering of global and local TCR:pMHC context. Global TCR space is represented by 2D projections of various TCR sequences, where antigen-specific TCRs are radially distributed about a pMHC and overlap designates TCR cross-reactivity. The local pMHC-specific TCR space shows the distinction between observed TCRs and ground truth TCRs in relation to model generations. (b) Probability mass function (PMF) of Equation (2) plotted for different values of ground truth TCRs and different model accuracies. K is fixed to be the current maximum of known TCRs for a given antigen $\approx$ 16,000. (c) Expected value of the F1 score is plotted for different average model accuracies given θ (the PMF simplifies to the conditional hypergeometric distribution). Red arrows indicate the percentiles of reference TCR counts (K) from real data.

A.3 Choice of Decoding Algorithm

While the architecture of the model and its training parameters directly influence the model's capacity to learn a robust conditional probability distribution over the TCR sequence space for a given pMHC, the actual generated sequences are derived from the algorithm or decision process by which tokens sampled from the learned distribution. Broadly there exist two classes: autoregressive and non-autoregressive sampling. The former, which is used in this work samples tokens y_t at time step t one at a time and conditions on the input x as well as the in-progress sequence generation: $y_t \sim P(y_t|y_{<t}, x, \theta)$, given source sequence x and model parameters θ. As such, while we can increase performance by changing the underlying model's learned distribution by experimenting with training methods, we also stand to make gains by tuning the method of sequence generation. An illustrated explanation of some commonly used decoding methods including those used here can be found at: `https://huggingface.co/blog/how-to-generate`.

We explore choices of sampling algorithms and further discuss their impact on the observed characteristics of the resulting translations in [11]. In this work, we selected beam search for all sequence generations, given its marked performance in the NLP setting as well as our proof-of-concept exploration. An important exception is the calculating the dataset-level Char-BLEU, where we used greedy decoding in line with previous work in NLP. Both decoding methods and the broader class of mode-seeking methods, aim to maximize for the highest probability conditional target sequence. A desirable property of mode seeking algorithms is that they are deterministic. Recently, however, mode-seeking algorithms have come under scrutiny for sampling only a small portion of the true target distribution, as noted in [12]. Instead of sampling tokens directly from the whole conditional distribution: $y_t \sim P(y_t|y_{<t}, x, \theta)$, mode-seeking algorithms try to approximate the $y^{MAP} = \arg\max_{y \in Y} \log p(y|x, \theta)$ which explores the conditional distribution about the mode. However, combined with the ability to assess and maintain longer high probability subsequences, the use of beam search results in a powerful method for sampling native sequence-like predictions, which we observed to be true for the TCR space [11, 13, 14].

A.4 Hyperparameter Optimization

Though both the BART and T5 models come with off-the-shelf recommendations for configurations that are empirically well suited for a number of NLP tasks, we sought to find the set of parameters best suited for the reduced vocabulary size and more tightly bounded sequence lengths when compared to natural language tasks. The impact of scaling in data constrained settings is marginal as shown by recent studies [15] that demonstrate the interplay between the data and model size. Even in this well defined setting, the number of parameters to tune is considerably large so we use a grid-based sweep to determine an optimized set of hyperparameters for the BART and T5 architectures for our case.

We first investigated the impact of coarse-grained choices in the larger model such as the width and depth by sweeping over model architecture and training algorithm values. For the model architecture we varied the number of attention heads, batch size, d_{model}, feed forward layer dimension, and number of total layers. All models were trained using the cross entropy loss with the AdamW optimizer. For the optimizer, we varied the learning rate and weight decay parameters. To compare model parameters from both the zero pre-training and pre-training+finetuning regimes, we ran a sweep on samples of the 'monolingual' (unlabeled TCR and pMHCs) and 'parallel' (paired TCR:pMHC) corpuses to tune performance on the pre-training and seq2seq task, respectively. Due to time and compute constraints, the sweeps were performed on a reduced sample of 100k TCRs and 100k pMHCs from the monolingual texts for pre-training. Since the parallel corpus was many orders of magnitude smaller to begin with, we took all the pairs not derived from the MIRA dataset (100k paired TCR:pMHC examples), as no allele imputation was used. Interestingly, we found that while the optimal configurations for the BART models were relatively consistent, the optimal T5 configurations for pre-training was a deeper, more narrow network and the one for direct training was a wider more shallow network (Figure S2a-b). To reconcile these differences for a single, task-agnostic, set of optimal parameters, we adopted the following heuristic: if the values were close, the one that resulted in an overall higher parameter count was chosen whereas if the values were far, an intermediate value was chosen. Slight adjustments were performed at the layer count level to adjust for parameters and make TCRBART and TCRT5 comparable. These values are reported in (Table S1). Surprisingly, we found that smaller models yielded better performance, deviating from the hyperparameters used in our previous works as well as the original BART and T5 papers. The final TCRBART architecture

uses 6 encoder and decoder layers at $d_{model} = 768$, totaling around 46 million parameters, while TCRT5 implements $d_{model} = 256$ and 10 encoder and decoder layers for a total of 42M parameters.

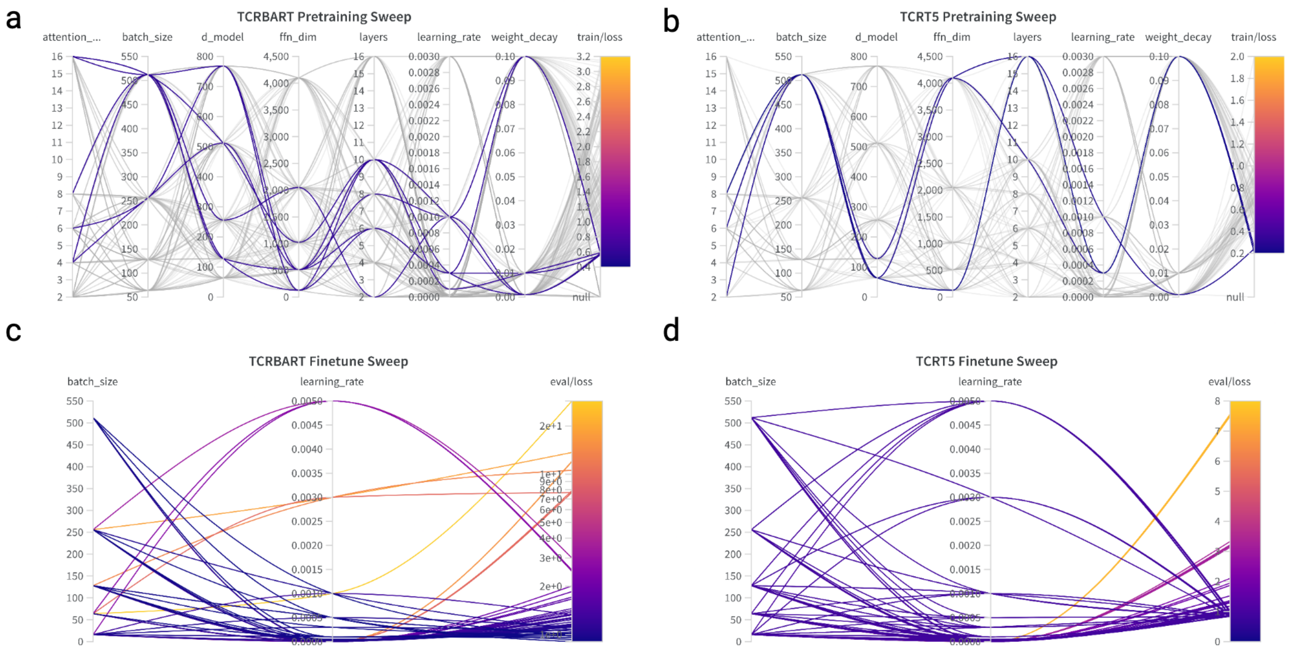

Supplementary Figure 2: Hyperparameter Optimization of TCRBART and TCRT5. Parallel coordinate plots showing the pretraining and finetuning parameters along with their corresponding training loss for pretraining (a-b) and finetuning (c-d). Pretraining was run for 3 epochs on the reduced set while finetuning was done for 5000 steps.

	TCRBART	TCRT5
Parameters	46M	42M
d_{model}	768	256
Vocab Size	28	128
Encoder Layers	6	10
Decoder Layers	6	10
Max Position Embedding	512	512
Attention Heads	16	16
Feed Forward Dim	128	1024
Cross Attention	✓	✓

Supplementary Table 1: Model Architecture Hyperparameters

A.5 Evaluation Methodology

Evaluating the performance of generative models for biological sequences, such as TCR sequences, poses unique challenges. Similar to much of NLP, especially machine translation, we build our framework around sampling known or ground truth target (CDR3β) sequences from experimental data. Due to the high cost of *in vitro* validation, assessing the fidelity and usefulness of generated sequences beyond these known sequences is difficult, particularly for de novo sequences. When even the most well represented pMHCs have on the order of 10^4 experimentally validated TCRs out of the theoretical 10^6, traditional accuracy and recall-based metrics often underestimate performance, inadvertently testing similarity to an arbitrary sample of validated examples (Figure S1).

In natural language processing, model-based scores such as BLEURT [16] and COMET [17] have been used as a means of scoring generated outputs when data generation and human evaluation are expensive or time consuming. Similarly, while one could use existing TCR-epitope recognition predictors to evaluate the antigen-specificity of the models' generations, their known issues with out of distribution generalization [18–20] risk introducing an opaque bias that is additionally confounded by these models' varying training data. In the antibody space, binding affinity between the antibody and the target is known to be one of the critical determinants of functional activity [21]. As such, accurate methods of *in silico* binding affinity prediction have been used to evaluate and even optimize de novo antibodies on their antigen-specificity [22, 23]. However, such methods of *in silico* evaluation of TCR:epitope specificity are less meaningful, since unlike antibodies, binding affinity and structural fit alone do not predict functional response of TCRs [24–26].

A.6 Checkpoint Selection

In deciding to choose which checkpoints to use for each model, we observed a marked difference between the performance dynamics of the models with and without pre-training. This is most clearly observed when plotting the diversity and accuracy metrics for each of the model checkpoints, showing distinct training trajectories in the utility space (Figure S3a-b). The pre-trained models demonstrate asymptotically increasing model performance across checkpoints for both the F1@100 and native sequence recovery metrics while the models that were trained directly from random initialization showed signs of potential overfitting, as both metrics peaked early on during training and dropped over additional iterations (Figure S3c-d). This was observed on reduced learning rates as well, indicating a possible regularization effect from pre-training [27]. A distinct difference between TCRBART and TCRT5 variants was the effect of including pre-training. While TCRT5 showed a significant improvement given pre-training, TCRBART showed worse performance. However, the finetuning's performance dynamics proved to be more stable than the non-pre-trained version, complicating the benefit of adding pre-training to TCRBART. Additionally we examined the number of unique sequences for the models over the checkpoints and saw that the TCRBART-0 and TCRT5-FT showed increasing number of unique sequences over training steps (Figure S3e). For each of the models, the checkpoint with the best performance on F1 was chosen to characterize the optimal performance from each model variant and assess their comparative advantages.

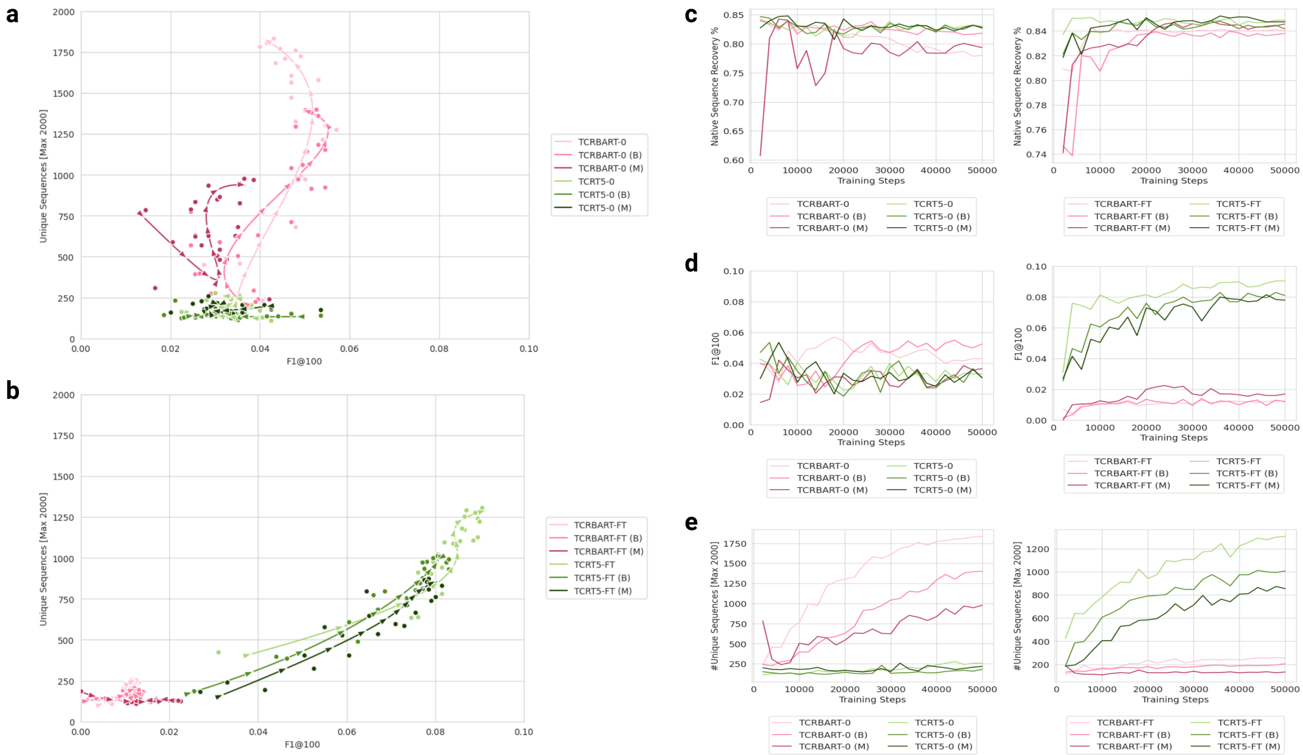

Supplementary Figure 3: Training dynamics highlight the robustness of pretrained models across checkpoints. Diversity vs. accuracy (F1) plotted for model checkpoints with smoothed interpolated splines and associated arrows showing the direction of model checkpoints through their training trajectory for: (a) Randomly initialized models (zero pre-training) (b) Pre-trained and finetuned models (c) Native sequence recovery for each checkpoint, colored by model, with panel split by pre-training status. (d) F1@100 for all checkpoints by pretraining status. (e) Number of unique generations for each checkpoint across training for all models with panel split by pre-training status. All models checkpoints were taken every 2000 steps across 20 epochs.

A.7 TCRT5 Data Ablation

After having identified TCRT5-FT as the best model, we ventured to check if we were using the best version of the data to train the model. To this end, we conducted an ablation study by removing key complexities of our training and data pipelines and measuring their effects on model performance. We started with our chosen model, TCRT5-FT, finetuned on the single-task TCR generation with semi-synthetic MIRA [28] data. Next, we retrained the model without the MIRA data for an equivalent number of steps to assess the contribution of the MIRA data, which has many representative TCRs per pMHC but a much narrower distribution centered around one disease context than the rest of the data. Finally, we removed pre-training altogether, training a model on the reduced dataset from random initialization to see if a TCRT5-0 model could outperform TCRT5-FT if the MIRA data was taken out.

To avoid over-representing the performance of the model trained on MIRA data on similar validation examples, we specifically removed three pMHCs that were a single edit distance from a MIRA example with a greater than 5% overlap in their cognate CDR3β sequences (LLLDRLNQL, TTDPSFLGRY, YLQPRTFLL) from the validation set. For all models, we used the same checkpoint heuristic, selecting the model with the highest F1 score. The results of these ablations are summarized in Table S2. We observed for the most part, each subsequent ablation resulted in a strictly worse performance with the notable exception that including the MIRA dataset caused a reduction in sequence diversity, as the TCRT5-FT (-MIRA) model, trained on a smaller but more diverse dataset, generated significantly more unique sequences. We include the values from our 12 model bake-off evaluated on all 20 validation pMHCs as a point of reference.

Model	Char-BLEU (↑)	F1@100 (↑)	%Rec. (↑)	mAP (↑)	N_{unique} (↑)
TCRBART-0	**93.5**	**.057**/.010	81.7/81.5	0.163	1276
TCRBART-0 (B)	93.0	.055/.015	81.9/83.9	**0.196**	**1302**
TCRBART-0 (M)	91.7	.042/**.030**	**84.3/85.9**	0.140	240
TCRBART-FT	87.4	.013/0.00	84.1/85.1	0.049	**240**
TCRBART-FT (B)	**89.8**	.014/**.010**	83.5/84.6	**0.062**	185
TCRBART-FT (M)	86.1	**.023/.010**	**84.5/85.0**	0.048	127
TCRT5-0	**89.5**	.040/.030	83.2/84.7	0.142	129
TCRT5-0 (B)	34.8	**.054/.035**	84.5/**85.9**	**0.167**	139
TCRT5-0 (M)	37.8	**.054**/.020	**84.7/85.9**	0.129	**177**
TCRT5-FT	**96.4**	**.091/.020**	**84.9**/85.0	0.246	**1300**
TCRT5-FT (B)	93.5	.083/.015	84.6/**85.1**	**0.279**	933
TCRT5-FT (M)	94.4	.082/**.020**	**84.7**/84.6	0.180	833
TCRT5-FT (+MIRA)	**97.1**	**.050/.020**	**83.8/84.9**	**0.207**	1044*
TCRT5-FT (-MIRA)	90.1	.027/.010	72.4/74.2	0.168	**1666***
TCRT5-0 (-MIRA)	79.4	.044/0.00	65.1/65.6	0.170	1060*

Supplementary Table 2: TCRT5 Ablation Study. Mean/Median values are reported where applicable. Best in class metric is highlighted in bold. Evaluation figures from our 12 model bake-off evaluated on all 20 validation pMHCs are shown as a point of reference. Data ablation models marked with an * were evaluated on 17 validation pMHCs (max 1700 unique sequences).

A.8 Revisiting Beam search for CDR3β sequences

Beam search is commonly used in sequence generation tasks for natural language processing (NLP) for a number of reasons. In addition to being deterministic, it has been empirically observed that asking the model to choose high-probability sequences often gives a more accurate, albeit shorter and less complex output. Unlike greedy decoding, which selects the most probable token at each step of decoding, or ancestral sampling, which draws from the multinational target distribution conditioned on the input and the previous tokens, beam search maintains multiple partially-constructed candidate sequences ("beams") simultaneously. At each sequence generation step, beam search considers all possible next tokens for each sequence in the current beam. It typically scores each potential

continuation ("hypothesis") by multiplying the cumulative sequence probability with the probability of the new token, creating a pool of new candidate sequences. From this pool, the top-k highest scoring sequences (k=number of beams) to continue to the next step. This process repeats until all candidates reach an end token or maximum length, maintaining at most the top-k candidates at each step (less if a sequence has reached its end). The outputs are typically returned in the order of score (sequence likelihood) with the highest-scoring complete sequence, being returned first. k=1 is a special case of the beam search algorithm: greedy decoding.

In the context of TCR sequence generation, beam search was observed to favor sequences with common motifs and high generation probabilities (high OLGA p_{gen} values). This behavior can be beneficial for pushing the generation of biologically plausible sequences. However, the way that beam search is implemented, the tradeoff for diversity becomes apparent as beam search samples locally around high probability prefixes. This explains why the motif 'CASS' was almost ubiquitously observed by TCRT5. In practice, this reduction in diversity results in potentially missing rare but functional TCRs that exist in natural repertoires, with strong reactivity to the target antigen. This property helps explain why beam search-generated sequences often showed higher average generation probabilities than reference repertoires, effectively sampling from a more conserved region of the vast TCR sequence space, recapitulated through sampling by model logits. It is noteworthy, however, that ER-TRANSFORMER+ achieved some lower p_{gen} sequences given this.

A.9 Metric Correlation

When we expanded out the twelve models' performance by pMHC, we noticed that the models' performance was largely conserved, indicating that some pMHCs were predisposed to higher performance than others (Figure S2a-b). We investigated the correlation of two of our performance metrics (F1@100 and sequence recovery) against features of our validation examples to see if we could find any associations between performance and characteristics of the data, specifically focusing on features that we felt had the potential to influence performance: the similarity to a training set pMHC, the target overlap with the closest known pMHC, and the number of known binders to the validation pMHC (Figure S2c). Of these comparisons, we found that as the number of references increased, the sequence recovery scaled accordingly (Spearman's ρ=0.69). F1, on the other hand had a slightly noisier relationship with the potential explanatory features with target set size being the strongest correlate (Spearman's ρ=0.58). In general, we observed that the examples with a larger number of reference target sequences had a greater sensitivity to a nonzero F1 score. We then proceeded to check the correlation between F1 score and the other metrics (Figure S2c-d). Of these, we observed a dynamic range of average F1 scores when the mean sequence recovery for an input reached 90%. To test if this was true on an individual sequence level, we evaluated the sequence recovery of all of the translations across all of the models after taking out exact matches from the reference set and saw if there was any separation between the sequence recoveries of known binders and those of unknown specificity. To our surprise, the threshold of 90% persisted and we saw a strong separation where known binders shared at least a 90% to another CDR3β sequence in the reference target set for a particular pMHC (Figure S2e). We posit that the 90% sequence identity to known binders may serve as a potentially useful proxy for F1 score in cases where few target sequences exist for an input pMHC and note a very similar finding on a similar edit distance variability around certain CDR3β and CDR3α motifs for a particular epitopes [29].

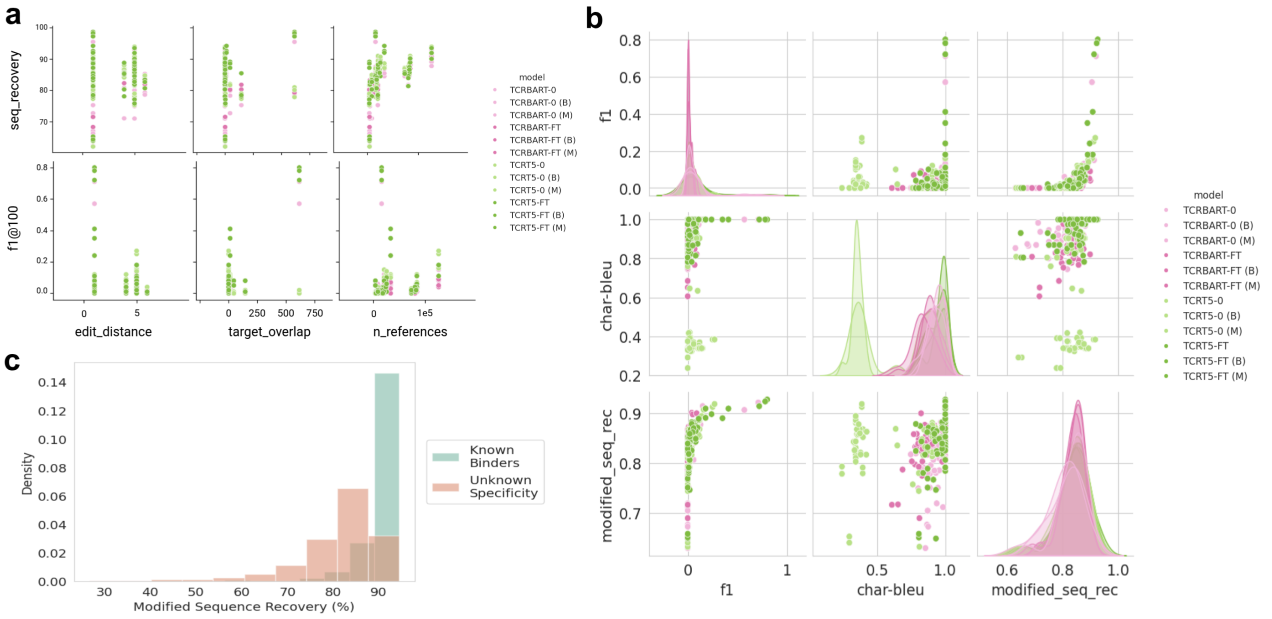

Supplementary Figure 4: Metric Correlation. (a) Scatterplot showing relationship between accuracy metrics (sequence recovery and F1@100) and input features (edit distance to closest training pMHC, TCR overlap with closest training pMHC (by edit distance), and number of references (known TCR binders). (b) Correlation plot between sequence-derived metrics. Pairplot showing the pairwise relationships of F1@100, Char-BLEU, and (modified) sequence recovery, across model variants. Modified sequence recovery is calculated by first removing exact matches to the generated sequences from the reference sets and calculating sequence recovery to the closest sequence. (c) Histogram of modified sequence recovery values stratified by known binding status.

A.10 Functional Validation of Computationally Designed TCR Sequences

Traditional TCR validation approaches often rely on peptide-MHC (pMHC) multimer binding assays to demonstrate antigen specificity. However, multimer binding presents several fundamental limitations that may not accurately reflect in vivo TCR functionality [30]. The primary concern with multimer-based detection lies in the disconnect between binding affinity and functional activation, as demonstrated by studies showing that TCRs capable of multimer binding do not always trigger downstream T cell activation [31]. This binding-function disconnect occurs because functional T cell activation requires not only initial TCR:pMHC interaction but sufficient dwell time and sustained signaling by kinetic proofreading that affinity based multimer assays cannot adequately capture [32].

Given these limitations, we elected to pursue functional validation using a luciferase-based T cell activation assay to assess whether computationally designed TCR sequences could mediate epitope-specific cellular responses that more closely approximate physiological T cell function. Our validation approach uses two key players: engineered T cells from an immortalized cell line (Jurkat cells) and modified antigen presenting cells that have lost the ability to process and present endogenous peptides (T2 cells). These Jurkat cells specifically have two modifications that make them quite useful for this experimental setup: First, they are TCR knockout cells meaning they don't express native TCRs, making them an ideal candidate for expressing TCRs. Second, these engineered Jurkat cells also come with a luciferase reporter under control of a nuclear factor of activated T cells (NFAT)-responsive promoter. This allows us to measure with minimal effort T cell activation, not just binding, by transducing our constructs into different pools of Jurkat cells co-cultured with antigen presenting cells and seeing which Jurkats light up, and how much. The T2 cells play a critical role here by ensuring that only the query peptide is presented. By inhibiting the endogenous peptide transport and presentation, a sufficient concentration of pulsed peptide can displace whatever is presented on the surface dwelling MHC molecules and replace it with near certainty. This provides us a clever system with a convenient readout that enables rapid functional validation of designed TCRs.

This functional approach offers several advantages over multimer-based validation. The assay measures actual T cell activation rather than just physical binding, providing physiologically relevant data about TCR function. The quantitative luciferase output enables assessment of activation strength across a dynamic range, capturing both weak and strong functional interactions. Additionally, the high-throughput nature of luciferase-based assays facilitates simultaneous testing of multiple constructs, enabling more comprehensive validation of computationally designed sequences. Future validation efforts would benefit from incorporating orthogonal functional assays such as cytokine production measurements and proliferation assays to provide more direct evidence of not only T cell activation but also effector function which is ultimately the desired outcome for these designer therapeutics. Similarly, conducting these experiments with primary T cells would offer more physiologically relevant contexts for assessing our framework. Structural validation of TCR:pMHC interactions through techniques such as surface plasmon resonance or crystallography would provide mechanistic insights into the molecular basis of observed functional activities as well as important measurements on kinetics. Finally, before this technology would be fit for clinical trials, comprehensive cross-reactivity screening would need to be conducted to better evaluate safety profiles for potential therapeutic applications.

A.11 Dataset Composition

One of the key difficulties in training a seq2seq model on TCR:pMHC is the imbalance of the labeled examples between pMHCs. Testament to how asymmetrically distributed the known antigen-specific TCR space is, our validation set of 20 individual pMHCs possess 68k non-redundant CDR3β sequences, which is over 80% of all the known TCR:pMHC pairs outside of SARS-CoV2 data (Figure S5a). These TCR:pMHC pairs, among the rest, have been identified using assay methods of varying confidence, sometimes with support from more than one assay method, increasing the confidence that a particular TCR confers some measure of true biological activity against a given pMHC, though this is seldom the case for the more sparsely sampled pMHCs (Figure S5b). For example the field's heavy usage of the MHC multimer (tetramer, pentamer, dextramer, etc.) as a high throughput and cost-effective method of determining TCR:pMHC interaction has been brought into question with an interesting recent discovery [31]. In addition, these various methods have differing noise profiles which in turn yield a highly diverse set of experimental conditions in which a TCR:pMHC interaction was labeled positive. Finally, there is the question of how the models pick up

HLA information if at all. With most of the data coming from HLA-A2 alleles, whether or not these models will generalize outside a narrow distribution of HLA alleles is something that remains to be seen (Figure S5c).

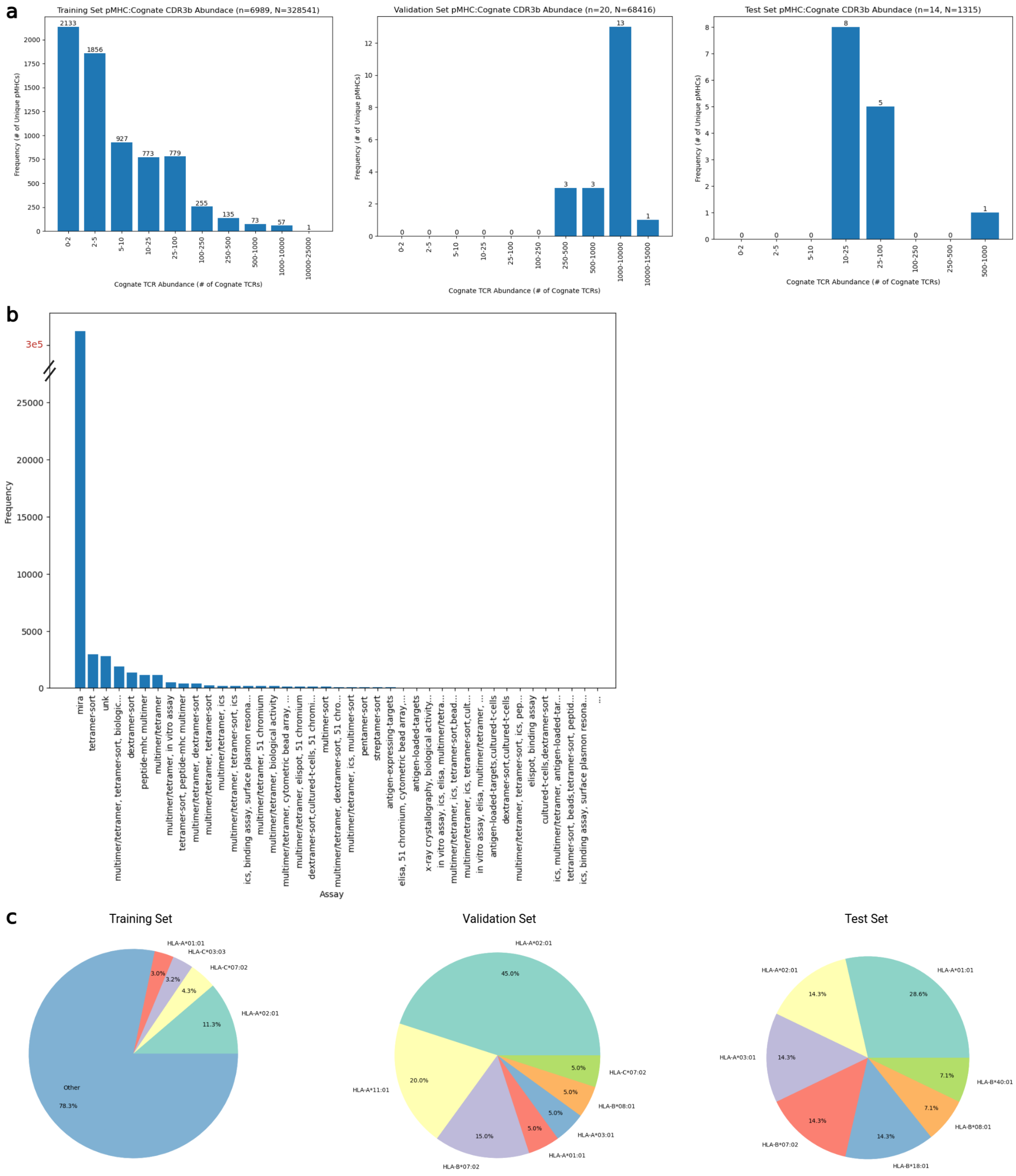

Supplementary Figure 5: TCR-TRANSLATE Dataset Statistics. (a) Distribution of pMHCs by cognate TCR count for training, validation, and test set. (b) Training set assay composition. (c) Allele breakdown of training, validation, and test set.

References

[1] Mike Lewis, Yinhan Liu, Naman Goyal, Marjan Ghazvininejad, Abdelrahman Mohamed, Omer Levy, Ves Stoyanov, and Luke Zettlemoyer. Bart: Denoising sequence-to-sequence pre-training for natural language generation, translation, and comprehension, 2019.

[2] Colin Raffel, Noam Shazeer, Adam Roberts, Katherine Lee, Sharan Narang, Michael Matena, Yanqi Zhou, Wei Li, and Peter J. Liu. Exploring the limits of transfer learning with a unified text-to-text transformer, 2020.

[3] Pranav Rajpurkar, Jian Zhang, Konstantin Lopyrev, and Percy Liang. Squad: 100,000+ questions for machine comprehension of text, 2016.

[4] Alex Wang, Amanpreet Singh, Julian Michael, Felix Hill, Omer Levy, and Samuel R. Bowman. Glue: A multi-task benchmark and analysis platform for natural language understanding, 2019.

[5] Jacob Devlin, Ming-Wei Chang, Kenton Lee, and Kristina Toutanova. Bert: Pre-training of deep bidirectional transformers for language understanding, 2019.

[6] Alec Radford and Karthik Narasimhan. Improving language understanding by generative pre-training. 2018. URL `https://api.semanticscholar.org/CorpusID:49313245`.

[7] Zihao Fu, Wai Lam, Qian Yu, Anthony Man-Cho So, Shengding Hu, Zhiyuan Liu, and Nigel Collier. Decoder-only or encoder-decoder? interpreting language model as a regularized encoder-decoder, 2023.

[8] Ashish Vaswani, Noam Shazeer, Niki Parmar, Jakob Uszkoreit, Llion Jones, Aidan N. Gomez, Lukasz Kaiser, and Illia Polosukhin. Attention is all you need, 2023.

[9] Don Mason. A very high level of crossreactivity is an essential feature of the t-cell receptor. *Immunology Today*, 19(9):395–404, 1998. ISSN 0167-5699. doi: https://doi.org/10.1016/S0167-5699(98)01299-7. URL `https://www.sciencedirect.com/science/article/pii/S0167569998012997`.

[10] Andrew Sewell. Why must t cells be cross-reactive? *Nature reviews. Immunology*, 12:669–77, 08 2012. doi: 10.1038/nri3279.

[11] Dhuvarakesh Karthikeyan, Colin Raffel, Benjamin Vincent, and Alex Rubinsteyn. Conditional generation of antigen specific t-cell receptor sequences. In *NeurIPS 2023 Generative AI and Biology (GenBio) Workshop*, 2023. URL `https://openreview.net/forum?id=SckdgVW3Kq`.

[12] Bryan Eikema and Wilker Aziz. Is map decoding all you need? the inadequacy of the mode in neural machine translation, 2020.

[13] Zhenghong Zhou, Junwei Chen, Shenggeng Lin, Liang Hong, Dong-Qing Wei, and Yi Xiong. Gratcr: Epitope-specific t cell receptor sequence generation with data-efficient pre-trained models. *IEEE Journal of Biomedical and Health Informatics*, 29(3):2271–2283, 2025. doi: 10.1109/JBHI.2024.3514089.

[14] Jiannan Yang, Bing He, Yu Zhao, Feng Jiang, Zhonghuang Wang, Yixin Guo, Zhimeng Xu, Bo Yuan, Jiangning Song, Qingpeng Zhang, and Jianhua Yao. De novo generation of t-cell receptors with desired epitope-binding property by leveraging a pre-trained large language model. *bioRxiv*, 2023. doi: 10.1101/2023.10.18.562845. URL `https://www.biorxiv.org/content/early/2023/10/20/2023.10.18.562845`.

[15] Niklas Muennighoff, Alexander Rush, Boaz Barak, Teven Le Scao, Nouamane Tazi, Aleksandra Piktus, Sampo Pyysalo, Thomas Wolf, and Colin A Raffel. Scaling data-constrained language models. In A. Oh, T. Naumann, A. Globerson, K. Saenko, M. Hardt, and S. Levine, editors, *Advances in Neural Information Processing Systems*, volume 36, pages 50358–50376. Curran Associates, Inc., 2023. URL `https://proceedings.neurips.cc/paper_files/paper/2023/file/9d89448b63ce1e2e8dc7af72c984c196-Paper-Conference.pdf`.

[16] Thibault Sellam, Dipanjan Das, and Ankur P. Parikh. Bleurt: Learning robust metrics for text generation, 2020. URL `https://arxiv.org/abs/2004.04696`.

[17] Ricardo Rei, Craig Stewart, Ana C Farinha, and Alon Lavie. Comet: A neural framework for mt evaluation, 2020. URL https://arxiv.org/abs/2009.09025.

[18] Dan Hudson, Ricardo A Fernandes, Mark Basham, Graham Ogg, and Hashem Koohy. Can we predict t cell specificity with digital biology and machine learning? *Nature Reviews Immunology*, pages 1–11, 2023.

[19] Filippo Grazioli, Anja Mösch, Pierre Machart, Kai Li, Israa Alqassem, Timothy J O'Donnell, and Martin Renqiang Min. On tcr binding predictors failing to generalize to unseen peptides. *Frontiers in Immunology*, 13:1014256, 2022.

[20] Morten Nielsen, Anne Eugster, Mathias Fynbo Jensen, Manisha Goel, Andreas Tiffeau-Mayer, Aurelien Pelissier, Sebastiaan Valkiers, María Rodríguez Martínez, Barthélémy Meynard-Piganeeau, Victor Greiff, Thierry Mora, Aleksandra M. Walczak, Giancarlo Croce, Dana L. Moreno, David Gfeller, Pieter Meysman, and Justin Barton. Lessons learned from the immrep23 tcr-epitope prediction challenge. *ImmunoInformatics*, 16, Dec 2024. ISSN 2667-1190. doi: 10.1016/j.immuno.2024.100045. URL https://doi.org/10.1016/j.immuno.2024.100045.

[21] Herman N Eisen and Gregory W Siskind. Variations in affinities of antibodies during the immune response. *Biochemistry*, 3(7):996–1008, 1964.

[22] Sharrol Bachas, Goran Rakocevic, David Spencer, Anand V. Sastry, Robel Haile, John M. Sutton, George Kasun, Andrew Stachyra, Jahir M. Gutierrez, Edriss Yassine, Borka Medjo, Vincent Blay, Christa Kohnert, Jennifer T. Stanton, Alexander Brown, Nebojsa Tijanic, Cailen McCloskey, Rebecca Viazzo, Rebecca Consbruck, Hayley Carter, Simon Levine, Shaheed Abdulhaqq, Jacob Shaul, Abigail B. Ventura, Randal S. Olson, Engin Yapici, Joshua Meier, Sean McClain, Matthew Weinstock, Gregory Hannum, Ariel Schwartz, Miles Gander, and Roberto Spreafico. Antibody optimization enabled by artificial intelligence predictions of binding affinity and naturalness. *bioRxiv*, 2022. doi: 10.1101/2022.08.16.504181. URL https://www.biorxiv.org/content/early/2022/08/17/2022.08.16.504181.

[23] Kevin Michalewicz, Mauricio Barahona, and Barbara Bravi. Antipasti: interpretable prediction of antibody binding affinity exploiting normal modes and deep learning. *bioRxiv*, 2023. doi: 10.1101/2023.12.22.572853. URL https://www.biorxiv.org/content/early/2023/12/23/2023.12.22.572853.

[24] Nishant Kumar Singh, Timothy P. Riley, Sarah Catherine B. Baker, Tyler Borrman, Zhiping Weng, and Brian M. Baker. Emerging concepts in tcr specificity: Rationalizing and (maybe) predicting outcomes. *The Journal of Immunology*, 199:2203 – 2213, 2017. URL https://api.semanticscholar.org/CorpusID:5375575.

[25] K. Christopher Garcia, Massimo Degano, Larry R. Pease, Mingdong Huang, Per A. Peterson, Luc Teyton, and Ian A. Wilson. Structural basis of plasticity in t cell receptor recognition of a self peptide-mhc antigen. *Science*, 279(5354):1166–1172, 1998. doi: 10.1126/science.279.5354.1166. URL https://www.science.org/doi/abs/10.1126/science.279.5354.1166.

[26] Xiang Zhao, Elizabeth M. Kolawole, Waipan Chan, Yinnian Feng, Xinbo Yang, Marvin H. Gee, Kevin M. Jude, Leah V. Sibener, Polly M. Fordyce, Ronald N. Germain, Brian D. Evavold, and K. Christopher Garcia. Tuning t cell receptor sensitivity through catch bond engineering. *Science*, 376(6589):eabl5282, 2022. doi: 10.1126/science.abl5282. URL https://www.science.org/doi/abs/10.1126/science.abl5282.

[27] Dumitru Erhan, Yoshua Bengio, Aaron Courville, Pierre-Antoine Manzagol, Pascal Vincent, and Samy Bengio. Why does unsupervised pre-training help deep learning? *J. Mach. Learn. Res.*, 11:625–660, March 2010. ISSN 1532-4435.

[28] Jennifer N. Dines, Thomas J. Manley, Emily Svejnoha, Heidi M. Simmons, Ruth Taniguchi, Mark Klinger, Lance Baldo, and Harlan Robins. The immunerace study: A prospective multicohort study of immune response action to covid-19 events with the immunecode™ open access database. *medRxiv*, 2020. doi: 10.1101/2020.08.17.20175158. URL https://www.medrxiv.org/content/early/2020/08/21/2020.08.17.20175158.1.

[29] Andreas Mayer and Curtis G. Callan. Measures of epitope binding degeneracy from t cell receptor repertoires. *Proceedings of the National Academy of Sciences*, 120(4):e2213264120, 2023. doi: 10.1073/pnas.2213264120. URL https://www.pnas.org/doi/abs/10.1073/pnas.2213264120.

[30] Cristina Rius, Meriem Attaf, Katie Tungatt, Valentina Bianchi, Mateusz Legut, Amandine Bovay, Marco Donia, Per thor Straten, Mark Peakman, Inge Marie Svane, Sascha Ott, Tom Connor, Barbara Szomolay, Garry Dolton, and Andrew K. Sewell. Peptide–mhc class i tetramers can fail to detect relevant functional t cell clonotypes and underestimate antigen-reactive t cell populations. *The Journal of Immunology*, 200(7):2263–2279, 04 2018. ISSN 0022-1767. doi: 10.4049/jimmunol.1700242. URL https://doi.org/10.4049/jimmunol.1700242.

[31] Marius Messemaker, Bjørn P.Y. Kwee, Živa Moravec, Daniel Álvarez-Salmoral, Jos Urbanus, Sam de Paauw, Jeroen Geerligs, Rhianne Voogd, Ben Morris, Aurélie Guislain, Maike Mußmann, Yaël Winkler, Maxime Steinmetz, Matyas Iras, Eric Marcus, Jonas Teuwen, Anastassis Perrakis, Roderick L. Beijersbergen, Wouter Scheper, and Ton N. Schumacher. A functionally validated tcr-pmhc database for tcr specificity model development. *bioRxiv*, 2025. doi: 10.1101/2025.04.28.651095. URL https://www.biorxiv.org/content/early/2025/05/12/2025.04.28.651095.

[32] T W McKeithan. Kinetic proofreading in t-cell receptor signal transduction. *Proceedings of the National Academy of Sciences*, 92(11):5042–5046, 1995. doi: 10.1073/pnas.92.11.5042. URL https://www.pnas.org/doi/abs/10.1073/pnas.92.11.5042.
